# Nitric oxide function in atherosclerosis

**DOI:** 10.1080/09629359791875

**Published:** 1997-02

**Authors:** K. E. Matthys, H. Bult

**Affiliations:** University of Antwerp (UIA) Division of Pharmacology Wilrijk Antwerp B2610 Belgium

## Abstract

Atherosclerosis is a chronic inflammatory process in the intima of conduit arteries, which disturbs the endothelium-dependent regulation of the vascular tone by the labile liposoluble radical nitric oxide (NO) formed by the constitutive endothelial nitric oxide synthase (eNOS). This defect predisposes to coronary vasospasm and cardiac ischaemia, with anginal pain as the typical clinical manifestation. It is now appreciated that endothelial dysfunction is an early event in atherogenesis and that it may also involve the microcirculation, in which atherosclerotic lesions do not develop. On the other hand, the inflammatory environment in atherosclerotic plaques may result in the expression of the inducible NO synthase (iNOS) isozyme. Whether the dysfunction in endothelial NO production is causal to, or the result of, atherosclerotic lesion formation is still highly debated. Most evidence supports the hypothesis that constitutive endothelial NO release protects against atherogenesis e.g. by preventing smooth muscle cell proliferation and leukocyte adhesion. Nitric oxide generated by the inducible isozyme may be beneficial by replacing the failing endothelial production but excessive release may damage the vascular wall cells, especially in combination with reactive oxygen intermediates.


Mediators of Inflammation, **6**, 3–21 (1997)

### Nitric oxide function in atherosclerosis

K. E. Matthys and H. Bult[CA]

University of Antwerp (UIA), Division of Pharmacology, B2610 Wilrijk, Belgium

[CA]Corresponding Author
Tel: (+32) 3 820 27 38
Fax: (+32) 3 820 25 67
Email: bult@uia.ua.ac.be

**ATHEROSCLEROSIS is a chronic inflammatory process in the intima of conduit arteries, which disturbs the endothelium-dependent regulation of the vascular tone by the labile liposoluble radical nitric oxide (NO) formed by the constitutive endothelial nitric oxide synthase (eNOS). This defect predisposes to coronary vasospasm and cardiac ischaemia, with anginal pain as the typical clinical manifestation. It is now appreciated that endothelial dysfunction is an early event in atherogenesis and that it may also involve the microcirculation, in which atherosclerotic lesions do not develop. On the other hand, the inflammatory environment in atherosclerotic plaques may result in the expression of the inducible NO synthase (iNOS) isozyme. Whether the dysfunction in endothelial NO production is causal to, or the result of, atherosclerotic lesion formation is still highly debated. Most evidence supports the hypothesis that constitutive endothelial NO release protects against atherogenesis e.g. by preventing smooth muscle cell proliferation and leukocyte adhesion. Nitric oxide generated by the inducible isozyme may be beneficial by replacing the failing endothelial production but excessive release may damage the vascular wall cells, especially in combination with reactive oxygen intermediates.**

**Key words:** Atherosclerosis, Endothelial cell, eNOS, iNOS, Intimal thickening, Nitric oxide, Peroxynitrite, Superoxide anion

### Atherosclerotic Lesion Development

### The intima is the soil for atherosclerosis

Atherosclerotic lesions develop in the inner coat (or tunica intima) of the aorta, the large elastic arteries e.g. the carotid arteries and the arteries supplying the lower extremities, and the medium-sized muscular arteries, such as the coronary arteries. At birth, the intima consists solely of endothelial cells, but soon after birth focal and circumferential thickening occurs.[1] This spontaneously developing intima consists of smooth muscle cells, connective tissue and isolated macrophages, and is considered an adaptation to mechanical wall stress.[2] Although not pathologic at this stage, the thickened intima marks locations where atherosclerosis tends to develop later in life under the influence of atherogenic stimuli e.g. hypercholesterolaemia.

### Features of human atherosclerosis

Early atherosclerotic lesions are characterized by the deposition of lipids and the appearance of macrophages and T-lymphocytes in the intima. As macrophages and a few smooth muscle cells underneath the endothelial cells accumulate lipid, they acquire a 'foamy' appearance. Clusters of lipid-laden cells become macroscopically visible as fatty streaks.[3] Progressively, these flat, fatty lesions transform to raised fibrolipid plaques, as intimal smooth muscle cells proliferate and deposit extracellular matrix, mainly collagen. In a subsequent stage, the advanced lesion has a characteristic microanatomy with a core of extracellular lipid separated from the media by smooth muscle cells and covered at the luminal side by a thick fibrous cap. Surrounding the lipid core are lipid-filled foam cells. The ischaemia in the necrotic core initiates angiogenesis. This type of plaque may cause narrowing of the lumen once the compensatory vascular remodelling process which

© 1997 Rapid Science Publishers increases the external diameter of the vessel becomes exhausted. Only then, lesions become angiographically visible. The final stage, the complicated plaque, may arise either from fissure of the fibrous cap or from intra-plaque haemorrhage. The thromboembolic events following plaque fissure are a major cause of clinically manifest acute ischaemic syndromes. If the thrombus is not occlusive, it becomes incorporated into the plaque and is organized by invading macrophages and smooth muscle cells, thereby further compromising the lumen of the vessel. The sequence of fissure, thrombus formation, organization and incorporation into the plaque may occur repeatedly.

### Models of atherosclerosis and intimal thickening

*Models of atherosclerosis*

Current knowledge of the initiation of the atherogenic process is largely based on rabbit or primate models of hypercholesterolaemia, which may be diet-induced or genetically determined as in Watanabe heritable hyperlipidaemic (WHHL) rabbits. Hypercholesterolaemia provokes intravascular lipid infiltration leading to the formation of fatty streaks, which resemble early human lesions.[3] Protracted cholesterol feeding eventually results in advanced fibrolipid plaques containing necrotic debris, as in advanced human disease.

*Models of intimal thickening*

Intimal thickening can be induced experimentally by creating a modest mechanical injury of the smooth muscle cells of the media. The most extensively investigated model involves balloon denudation of the intima of the rat carotid artery with an embolectomy catheter.[4] The discrete mechanical injury of the underlying media evokes smooth muscle cell proliferation in the media, followed by migration to the intima and an extended phase of intimal proliferation. The endothelial cells are completely removed by the initial insult and regrowth of the endothelial cells from the lesion edges is virtually absent. The removal of the endothelial cells is not essential nor sufficient for the process of intimal hyperplasia.

Placing a flexible collar around the rabbit carotid artery does not create direct endothelial injury, but induces smooth muscle cell proliferation in the media, followed by migration and prolonged proliferation in the intima.[5] Both models illustrate the three wave paradigm for the involvement of smooth muscle cells in the formation of intimal cushions.[4]

The inflation of an angioplasty balloon in arteries of rabbits, pigs or other experimental animals is used to mimic restenosis due to accelerated intimal thickening after percutaneous transluminal coronary angioplasty (PTCA). The vessel wall distension by the repeated inflation of a slightly oversized balloon creates a much more extensive injury of the media and the lamina elastica interna than the gentle passage of an embolectomy catheter. Unlike balloon denudation, the balloon angioplasty thus predisposes to thrombus formation. In accordance with restenosis after PTCA in humans, the incorporation and organization of the non-occlusive thrombus adds to the bulk of neointima formation.[4] A further difference with balloon denudation is the quick and often complete recovery of the endothelial cell layer through outgrowth from patches of cells which remained present after the angioplasty.

### Atherosclerosis is a Chronic Inflammatory Process

The long-standing and continuously refined 'response-to-injury' hypothesis[6] considers the lesions as the result of an excessive inflammatory-fibroproliferative response to various forms of insults to the endothelium and smooth muscle. Moreover, the presence of T-lymphocytes in atherosclerotic lesions at all stages of development points to an important immunologic component in atherogenesis.[7] T-lymphocytes and macrophages are capable of producing numerous inflammatory mediators and growth factors, and have been demonstrated to be in an activated state in atherosclerotic lesions.

### Pathogenetic mechanisms in hypercholesterolaemia-induced atherogenesis

Several different sources of injury to the endothelium can lead to endothelial dysfunction and initiate the disease process.[6] In hypercholesterolaemia-induced atherosclerosis, the major causal agent is now assumed to be oxidized LDL (oxLDL).[8,9] Oxidation of lipoproteins flooding the intima may result from the production of reactive oxygen intermediates or 15-lipoxygenase activity in the endothelial cells (Fig. 1). OxLDL in turn is cytotoxic to endothelial cells by the metal-catalysed production of free radicals from lipid hydroperoxides contained in the modified lipoprotein particle.[10] Furthermore, oxLDL is chemotactic for monocytes and T-lymphocytes. Newly formed epitopes in oxLDL elicit cell-mediated and humoral immune re-

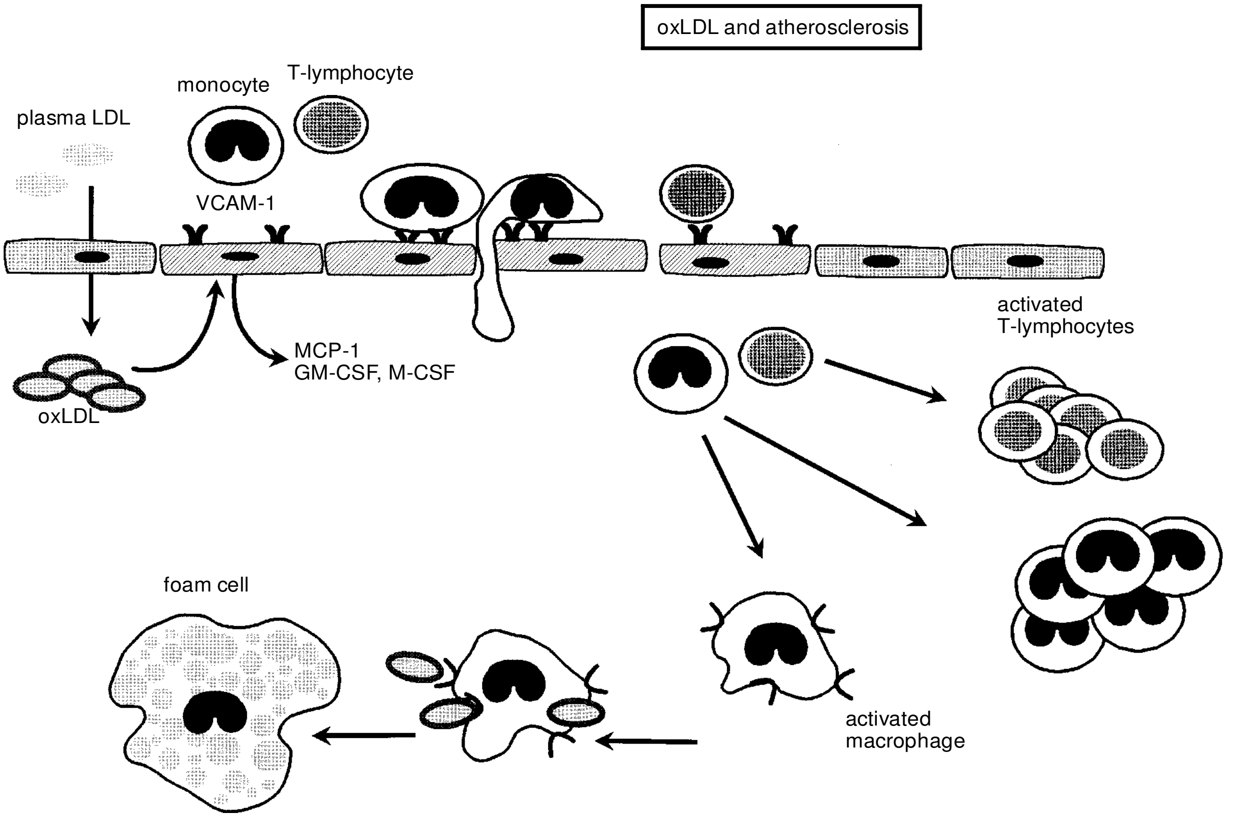

FIG. 1. Lipoproteins flooding the intima become oxidized. Oxidized LDL (oxLDL) is chemotactic for monocytes and T-lymphocytes. OxLDL promotes mononuclear cell adhesion, infiltration and proliferation by stimulating the endothelial cells to express adhesion molecules, e.g. VCAM-1, and to produce chemotactic factors, e.g. MCP-1, and growth factors. Uptake of oxLDL by macrophages leads to foam cell formation.

sponses.[7] Minimally oxidized LDL stimulates the endothelial cells and smooth muscle cells to secrete monocyte chemotactic protein-1 (MCP-1) and growth factors involved in the differentiation and proliferation of monocytes, and oxLDL may, synergistically with cytokines, promote mononuclear leukocyte adhesion to the endothelium through the induction of vascular cell adhesion molecule-1 (VCAM-1).[11,12] The oxidative stress-sensitive nuclear transcription factor κB (NF-κB) may be a crucial intermediate in the inflammatory activation of the endothelium.[13] Monocyte-derived macrophages internalize oxLDL through scavenger receptors. As these receptors are not down-regulated by the intracellular cholesterol level, massive cholesterol accumulation occurs and the macrophages transform to foam cells.

Thus, it appears that endothelial cells, through the oxidation of LDL, recruit macrophages to remove the invaded lipoprotein particles. This attractive hypothesis also implies that a chronic inflammatory response will develop if the macrophages are unable to eliminate oxLDL sufficiently.

### Atherosclerosis and Nitric Oxide Signalling

### The nitric oxide signalling pathway in normal arteries

A major step forward in the understanding of blood vessel physiology was the discovery by Furchgott and Zawadzki[14] of a factor released by the endothelium that relaxed the underlying smooth muscle. This endothelium-derived relaxing factor (EDRF) was later identified as nitric oxide (NO)[15–17] or a related nitrosylated compound e.g. S-nitrosocysteine.[18,19] Nitric oxide is formed by a five-electron oxidation of a terminal guanidino nitrogen atom of the amino acid L-arginine, with concomitant formation of L-citrulline, by an enzyme known as nitric oxide synthase (NOS).[20] There are two major classes of NO synthases: constitutive and inducible (iNOS) enzymes. Constitutive isoforms are expressed in endothelial cells (eNOS), in neuronal cells (nNOS) and in certain other cell types. The activity of these isoforms is strictly calcium-calmodulin dependent and is present both in the cytosol and associated with membranes.

Stimulation of the appropriate receptors on the endothelial cell by physical (shear stress resulting from increased flow, mechanical deformation) or chemical (acetylcholine, bradykinin, substance P, ATP) stimuli raises the cytoplasmic calcium levels, with concomitant eNOS activation and formation of NO.[21] Serotonin (5-hydroxytryptamine, 5-HT), although a potent vasoconstrictor through activation of 5-$HT_2$ receptors on the vascular smooth muscle cells, also mediates dilatation by the EDRF/NO-dependent mechanism, through activation of 5-$HT_1$-like receptors on the endothelium.[22] In biological systems, the dominant reactions of NO will be with another free radical such as superoxide anion, transition metals such as haem iron, or oxygen.[20] In the vessel wall, NO diffuses into the underlying smooth muscle cells to react with the haem group of a cytoplasmic guanylate cyclase. The formation of cyclic GMP then causes vasodilatation.[23–25]

Nitric oxide also raises cyclic GMP in the endothelial cells themselves, which inhibits the production of the potent endothelium-derived contracting factor endothelin.[26,27] Inhibitors of NOS and guanylyl cyclase revealed an inhibitory role of NO but not of cyclic GMP in endothelin secretion by porcine aortic endothelial cells.[28]

### Nitric oxide signalling in atherosclerosis

It has been recognized for a long time that atherosclerotic blood vessels are very susceptible to the development of vasospasm *in vivo*[29–32] and are hyperreactive to contractile agonists *in vitro*.[32–35] Because coronary vasospasm can be provoked by several stimuli with different mechanisms of action, it has been proposed that dysfunction or denudation of the endothelium in atherosclerosis may contribute to that phenomenon by leaving constrictor responses unopposed.[36–38] Even before it was realized that NO accounts for the biological activity of endothelium-derived relaxing factor, it was indeed demonstrated that artery segments obtained from atherosclerotic animals showed a loss of endothelium-dependent relaxation in organ bath experiments.[35,39–42] From then on, numerous *in vitro* studies confirmed the defect in the NO signalling pathway in isolated atherosclerotic blood vessels in rabbits,[43–49] pigs,[50–53] rats,[54] primates[55] and humans.[43,56–58] Basal as well as stimulated NO release appeared to be affected.[58–60]

Urinary nitrate, an index metabolite for NO formation *in vivo*,[61,62] is decreased in cholesterol-fed rabbits.[63] Catheterization-based studies in patients with coronary artery disease also demonstrated the impairment of endothelium-dependent coronary vasodilatation to acetylcholine[64–69] or increased flow,[70–72] particularly at atherosclerosis-prone branch points.[73] The deterioration of endothelium-dependent vasodilatation is an early event, as it can be observed in patients with typical angina or cardiac risk factors but with angiographically smooth coronary arteries.[73–80] The current weight of evidence suggests that impaired endothelium-dependent vasodilatation is the predominant mechanism underlying inappropriate constriction leading to ischaemic manifestations.[75,81,82] The instantaneous relief of the ischaemic attacks by the NO donor nitroglycerin points to a defect in the endogenous NO pathway. Unopposed vasoconstrictor responses in general, but also the loss of the EDRF-component in the net reaction to some agonists, e.g. serotonin and norepinephrine in the pig and dog,[37] and increased endothelin release in the absence of EDRF may contribute to the occurrence of vasospastic events in atherosclerotic vessels.

### The systemic nature of the defect in NO signalling

The EDRF/NO pathway is also active in the small vessels determining the resistance of the vascular tree,[83,84] thus contributing to blood pressure regulation.[85] Several studies (reviewed by Anderson *et al.*[86]) demonstrated that atherosclerosis in conduit vessels is accompanied by impaired endothelium-dependent vasodilatation in the microcirculation e.g. in the coronary[76,87–89] and peripheral resistance vessels.[90–93] Also the mere presence of cardiovascular risk factors was associated with dysfunctional microvascular endothelium.[94,95] While the expected hypertensive effect might contribute to the progression of cardiovascular disease, this also implies that, besides a dysfunctional endothelial NO pathway, other factors are involved in the initiation and progression of atherosclerotic plaques, since lesions do not develop in these microvessels. The systemic nature of the endothelial dysfunction could be of use in the non-invasive evaluation of endothelial function in readily accessible arteries.[96]

In summary, established atherosclerosis or the presence of risk factors e.g. hypertension, hypercholesterolaemia, and even male gender,[97,98] decrease the activity of the EDRF/NO pathway. Endothelium-dependent dilatation is lost progressively as atherogenesis continues. Conduit vessels with lesions as well as resistance vessels in which lesions do not develop are affected, the latter apparently to a lesser extent.[65,93]

### Explanations for the Defective NO Signalling Pathway

The mechanisms underlying the dysfunctional endothelial NO signalling pathway in atherosclerosis and hypercholesterolaemia are multifactorial (reviewed in Refs 99 and 100). Atherosclerotic arteries demonstrating disturbed endothelium-dependent relaxation are still capable of dilating to the NO donor nitroglycerin which provides the smooth muscle with NO upon enzymatic or thiol-dependent bioconversion.[101] As this demonstrates that the smooth muscle is still responsive to the dilatory action of NO, defective endothelial EDRF/NO release or increased NO inactivation after release appear to be involved. This is supported by the decreased release of bioactive NO from isolated perfused atherosclerotic arteries as assessed by a superfusion bioassay.[41,44,50,102,103]

### Endothelial receptor dysfunction

Endothelium-dependent dilatation is lost in a progressive, hierarchical fashion.[82] Vasodilator responses to acetylcholine and serotonin are lost early, before impairment of the dilatation to other receptor agonists e.g. substance P, to the receptor-independent stimulus calcium ionophore A-23178 or to mechanical stimuli. The agonist specificity of the early dysfunction suggests that it is not caused by a nonspecific impairment of the ability of the endothelial cells to produce NO, but points to selective alterations in endothelial receptor function or post-receptor effector pathways. This view is strengthened by the observation that the receptor-induced release of other endothelial products, such as prostacyclin, is attenuated as well.[104,105] In this respect, it has been demonstrated that the pertussis toxin-sensitive $G_i$ protein signalling pathway, which is employed by serotonin to elicit endothelium-dependent relaxation in the pig coronary artery, is impaired in the early stages of the atherosclerotic process.[106] Several studies have demonstrated that incubation of vessel segments with lipoproteins, and in particular with oxLDL, inhibited endothelium-dependent relaxation in a way similar to hypercholesterolaemia *in vivo* (reviewed by Flavahan[99]). Lysophosphatidylcholine, a component of oxLDL, mimicked the effects of the whole particle,[107–109] inhibited agonist-stimulated calcium signalling in cultured endothelial cells[110] and selectively inhibited $G_i$ protein-dependent signalling in porcine endothelial cells.[111]

### Expression of eNOS activity

The receptor selectivity (see above) argues against a reduced expression of eNOS activity in endothelial cells overlying atherosclerotic lesions. This assumption has recently been confirmed by *in situ* hybridization of eNOS mRNA and immunohistochemistry of eNOS protein in the aorta of hypercholesterolaemic rabbits. The results suggested that the expression of eNOS mRNA and protein was even increased in endothelial cells overlying fibrofatty plaques.[112] Studies of the expression and the activity of eNOS after *in vitro* exposure of endothelial cells to LDL, oxLDL or cholesterol yielded contradictory results, ranging from initial upregulation, via no effect to downregulation of eNOS expression. After acute exposure of the isolated rabbit carotid artery to cholesterol-rich liposomes, acetylcholine-induced release of EDRF/NO was evaluated functionally in a superfusion bioassay and appeared to be enhanced.[113] This could be due to augmentation of the release and/or prolongation of the half-life of NO. Exposure of endothelial cell membranes to liposomal cholesterol raised the activity of plasma membrane bound eNOS at low cholesterol concentrations, but had the opposite effect at higher concentrations.[114] The effects were attributed to modulations of the lipid environment of the membrane bound eNOS. Cholesterol was without effect on the activity of eNOS in the cytosol of endothelial cells. Interestingly, the increased activity of particulate eNOS was accompanied by a concentration-dependent increase in superoxide anion production, but the authors did not investigate whether eNOS was the source (see below). Low concentrations of oxLDL have been reported both to increase[115] and to decrease[116] the expression of mRNA, protein and activity of eNOS in cultured endothelial cells. The upregulation of eNOS mRNA was mimicked by lysophosphatidylcholine, one of the many constituents of oxLDL. This discrepancy between both reports could be due to large variability among different preparations of oxLDL with respect to biological activities. Downregulation of the expression of mRNA of eNOS by higher concentrations of oxLDL appears to be a more consistent finding.[115,116]

Native LDL was without effect on eNOS mRNA levels and eNOS activity,[115,116] although exposure of cultured endothelial cells to LDL may promote superoxide anion formation by eNOS[117] (see below).

### Arginine availability

Although arginine availability seems to be sufficient initially in view of the receptor-selectivity of the endothelial dysfunction (see above), studies on endothelium-dependent vasodilatation suggest that L-arginine depletion may occur. It should be noted, however, that the studies addressing the effect of the NO precursor show discordant results. In this respect, the behaviour of conduit arteries with overt atherosclerosis appears to be different from arterioles in the microcirculation, in which atherosclerosis does not develop.

### *Conduit arteries with atherosclerosis*

Most authors agree that *in vitro* L-arginine addition fails to restore the endothelium-dependent relaxations in the aorta[118–120] or femoral artery[121] with cholesterol-induced atherosclerotic lesions. One report showed that acute *in vivo* L-arginine administration to hypercholesterolaemic rabbits improved the endothelium-dependent relaxations in isolated large vessels *in vitro*, but it should be noted that responses to nitroglycerin were affected to a very similar extent.[122] Also prolonged *in vivo* L-arginine treatment ameliorated endothelium-dependent relaxations of isolated segments only marginally.[123] As the endothelial dysfunction is strictly dependent on the size of the lesions in rabbit conduit arteries,[35,49,124,125] the marked anti-atherogenic effect of prolonged L-arginine supplementation[123] (see below) most likely explains the improved endothelium-dependent relaxations. In patients with coronary or peripheral artery occlusive disease, a positive effect of L-arginine on endothelium-dependent dilatation of the conduit arteries was lacking.[126,127]

### *Conduit arteries without overt atherosclerosis*

Although the rabbit basilar artery develops neither atherosclerotic lesions nor a clear endothelial dysfunction after prolonged hypercholesterolaemia,[49] an improvement of the endothelium-dependent relaxations has been reported after *in vitro* exposure to L-arginine.[128] In addition, L-arginine also attenuated the augmented vasoconstrictor responses to potassium chloride, serotonin and endothelin. The authors suggested that the normalization by L-arginine of both the endothelium-dependent relaxation and the constrictor responses was the result of increased EDRF production. However, as cyclic GMP-mediated relaxation induced by endothelium-independent agonists was not studied, and as the contraction to a depolarizing potassium chloride solution is not affected by basal EDRF release,[37] it is not entirely clear whether the actions of L-arginine can be attributed solely to enhanced endothelial NO production. Lefer and Ma measured constrictions evoked by the NOS inhibitor L-NAME as an index of basal NO release by the endothelial cells of rabbit coronary arteries isolated after three weeks of cholesterol diet.[60] A reciprocal relationship existed between L-NAME evoked contractions and plasma cholesterol, suggesting that basal NO release by the segments became compromised in the absence of overt atherosclerosis. *In vitro* addition of L-arginine almost totally restored this index of basal NO production. However, as non-endothelial iNOS may be induced in arteries of cholesterol-fed rabbits,[129] it cannot be excluded that the vasoconstrictor responses to L-NAME resulted from inhibition of iNOS rather than eNOS.

### *Arterioles without overt atherosclerosis*

The results obtained in conduit arteries suggest that L-arginine may upregulate the impaired eNOS activity only if the cholesterol-exposed arteries are still lesion-free. Accordingly, all studies, except one,[130] reported that L-arginine infusion resulted in marked improvement to complete restoration of endothelium-dependent vasodilatation in the coronary and peripheral microcirculation in hypercholesterolaemic rabbits,[131] pigs[88] and humans.[126,132]

The mechanism of the amelioration of endothelium-dependent relaxations by L-arginine is not yet clear, and could be due to an interaction with smooth muscle cells or other effects. In view of plasma levels of L-arginine in the range of 150 to 250 μM and a $K_m$ of 5 to 10 μM for NOS isoforms, it is indeed surprising that L-arginine availability can ever limit NO biosynthesis.[20] L-arginine enters cells by facilitated diffusion via the $y^+$ transporter.[133] As exogenous L-arginine addition neither induces endothelium-dependent relaxations by itself, nor enhances agonist-induced endothelium-dependent relaxations in normal isolated vessel rings,[120,129,134] the intracellular stores appear to be sufficient for maximal eNOS activity in physiological circumstances. The increase in membrane cholesterol associated with hypercholesterolaemia might impair endothelial L-arginine transport, thus eventually depleting the intracellular stores. The latter may also result from the increased output of inactive nitrogen oxides, as demonstrated in hypercholesterolaemic rabbit aorta.[103] However, reversal by L-arginine of hypercholesterolaemic endothelial dysfunction may not simply reflect the replenishment of the substrate for NO production. The observation that the effect of L-arginine administration to hypercholesterolaemic rabbits is not sustained and depends on the anatomic site and sex[135] indeed supports a more complex mechanism of action.

As the best results are obtained in the microcirculation after L-arginine treatment *in vivo*, other less well characterized systemic effects of the amino acid e.g. its secretagogue effects on the adrenals and pituitary gland, may prevail.[133] This is illustrated by observations in healthy persons, where L-arginine infusion stimulated basal and acetylcholine-induced relaxation in the peripheral circulation[130,136,137] and decreased the systemic blood pressure.[127,138] The concomitant increase in urinary nitrate and cyclic GMP could not simply be attributed to a direct stimulating effect of L-arginine on eNOS, as prostaglandin $E_1$-induced dilatation also increased these parameters in the urine.[127] Furthermore, intravenous L-arginine administration increased urinary flow, which by itself resulted in enhanced excretion of nitrate and CGMP, in the absence of elevated nitrate plasma levels.[138]

### Endothelial NO synthase inhibition

L-arginine may be effective in conditions where endogenous NOS inhibitors are formed. $N^G$,$N^G$ dimethylarginine (DMA) has been found in the urine and plasma of humans and inhibited macrophage and vascular NO synthesis *in vitro* and *in vivo* in animals and humans,[139] suggesting the existence of endogenous mechanisms to regulate NO synthesis. Recently, DMA was reported to be increased in the serum of cholesterol-fed rabbits.[140,141] All classes of NO synthases are liable to feedback inhibition by NO,[142] probably by the interaction of NO with the haem prosthetic group.[143] Hence, high output NO production by iNOS (see below) might downregulate eNOS activity. This is supported by the observation that chronic *in vivo* administration of large doses of an NO donor to rabbits depressed the *ex vivo* output of EDRF/NO in response to acetylcholine, as assessed by means of bioassay.[135] Endothelial NOS may also be suppressed by other locally produced inflammatory mediators e.g. the T-lymphocyte-derived mediator interferon-$\gamma$.[144]

### Inactivation of NO by superoxide anion

Superoxide anion is known to inactivate EDRF/NO.[145,146] Generation of superoxide anion *in situ* in normal vessels reduced endothelium-dependent relaxation.[147,148] Under normal conditions, inactivation of EDRF by superoxide radicals is prevented by cytosolic CuZn superoxide dismutase (SOD)[149] and by extracellular SOD type C associated with heparan sulphate proteoglycans on the endothelial cell surface and in the interstitium.[148]

Hypercholesterolaemia in the rabbit increased the intimal[103,150] production of reactive oxygen species, resulting in increased degradation of NO (reviewed by Harrison and Ohara[100]). The tunica media beneath the atheromatous plaque in WHHL rabbits also inactivated EDRF/NO by an SOD-sensitive mechanism.[44] Increased vascular production of reactive oxygen species may result from enhanced xanthine oxidase activity in the endothelium[150] or from production by infiltrated monocytes.[151] In addition to direct inactivation of EDRF/NO by oxLDL and lysophosphatidylcholine,[152] oxLDL has been shown to stimulate the respiratory burst in neutrophils,[153] and lysophosphatidylcholine induced superoxide production in vascular smooth muscle cells via protein kinase C activation.[154] Endothelial NADPH oxidase systems,[155] activated by protein kinase C,[156] may also be involved. Protracted endothelial cell exposure to atherogenic native LDL concentrations increased superoxide anion production by three independent oxidative systems—cyclooxygenase, P450 isozyme and eNOS—of which the latter appeared to be the greatest source.[117] Nitrotyrosines, hallmarks of peroxynitrite formation from superoxide and NO, were detected intracellularly.

Furthermore, a striking feature of NOS is its ability to generate superoxide anion when either L-arginine or the cofactor tetrahydrobiopterin is limiting.[20] Under these circumstances, NADPH oxidation is uncoupled from synthesis of NO, and oxygen becomes the electron acceptor, resulting in superoxide formation. This has been demonstrated to occur in the constitutive NOS of the brain. Whether the low arginine levels needed for superoxide biosynthesis occur in intact endothelial cells *in vivo* is unclear. Arginine depletion of eNOS might occur from high local L-arginine consumption by iNOS (see below). Arginine availability may also be reduced by impediment of cellular uptake or delivery to eNOS, as has been suggested to occur in endothelial cell cultures treated with native LDL.[117] In the latter experiments, LDL-exposed cells produced significantly more superoxide anion than untreated cells, which was reversed by arginine supplementation. Inducible NOS did not seem to be involved, as $Ca^{2+}$-independent arginine-to-citrulline conversion under apparent $V_{max}$ conditions was low. Nevertheless, in conditions where iNOS, which is much more demanding for substrate then eNOS, is induced, insufficient L-arginine might result in superoxide anion release. This would also explain the benefit of providing L-arginine, i.e. to promote re-coupling, thus reducing vascular superoxide production and prolonging the half-life of EDRF/NO. These findings provide new insight into the mechanisms by which hypercholesterolaemia might both stimulate superoxide production and decrease functional NO levels.

The disturbed balance between vascular superoxide and endothelial nitric oxide production, resulting in the loss of functional NO, may be compensated for by iNOS activity in the vascular wall (see below) and/or by upregulation of endogenous SOD.[157–159] Addition in the organ bath of CuZn SOD, which does not penetrate cells, or preincubation with extracellular SOD type C, which binds extracellularly to vascular structures, also protected against the detrimental effects of superoxide radicals on endothelium-dependent relaxation.[148] Conversely, exhaustion of these protective mechanisms, which may be time-, species-, or vessel-dependent, may tip over the balance towards a net decrease in functional EDRF/NO.

In rabbits, but not in pigs, hypercholesterolaemia alone did not impair the endothelial dilator function in large vessels, but only occurred in arteries with intimal plaques,[35,44,46,48,49,125] with the exception of the coronary arteries.[60,160] Apparently, the rabbit is capable of keeping the superoxide and nitric oxide production in balance, as long as lesions do not develop. Superoxide production in the media beneath the plaque[44] or the presence of fatty streaks containing large amounts of macrophages and lipids, may disturb the balance by the high local superoxide production and the trapping of the lipophilic NO molecule.

Raising the antioxidant capacity in the vessel wall by the administration of CuZn superoxide dismutase, polyethylene-glycolated[161] or liposome-entrapped[121] to ensure cell entrance, partly restored the endothelium-dependent relaxation in the isolated aorta of the cholesterol-fed rabbit.[121,161] In keeping with these findings, it has been shown that addition of antioxidant vitamins in the diet of cholesterol-fed rabbits preserved the endothelium-dependent dilatation in the absence of an effect on lesion formation.[162,163] Also, dietary correction of hypercholesterolaemia in the rabbit normalized both the endothelial superoxide production and dramatically improved the vasodilator response to acetylcholine.[164] Oral administration of 2 g ascorbic acid produced marked improvement in the forearm vascular response to hyperaemia in patients with coronary artery disease.[165] However, short-term treatment with antioxidants of patients with hypercholesterolaemia did not improve the forearm vascular responses to acetylcholine.[166,167]

Eventually, atherosclerotic plaques, in particular when lipid-rich, may trap NO and may also mechanically disturb the normal dilatation of the medial smooth muscle. At this stage, the relaxation to exogenous NO donors e.g. nitroglycerin, and to endothelium-independent dilator substances e.g. atrial natriuretic peptide also becomes impaired.[102]

### Atherosclerosis and Inducible Nitric Oxide Synthase Expression

Animal and human macrophages,[168,169] smooth muscle cells[170–172] and endothelial cells[173,174] are capable of expressing iNOS after stimulation with endotoxin or cytokines. In contrast to eNOS, iNOS produces high amounts of NO for a sustained period.[20] In early reports, the presence of a constitutive NOS in vascular smooth muscle cells has been suggested,[175,176] but these observations were probably related to the induction of iNOS during the isolation and manipulation of the cells or tissue.[172,177,178]

### Atherosclerosis and iNOS

Only recently, functional and biochemical evidence suggested that cholesterol feeding of rabbits induced iNOS expression in the aorta[129] and in the lungs.[179] The addition of NOS inhibiting L-arginine analogues caused endothelium-independent contractions in the isolated atherosclerotic rabbit aorta, pointing to the continuous formation of NO by subendothelial iNOS.[129] The observation that the NOS inhibitors nitro-L-arginine methyl ester (L-NAME) and monomethyl-L-arginine (L-NMMA) were equipotent in this respect further supported the involvement of iNOS.[129] The expression of iNOS may account for the increased output of nitrogen oxides in arteries of cholesterol-fed rabbits.[103] Histochemical studies in WHHL rabbits confirmed the expression of iNOS in medial and intimal smooth muscle cells, and showed significant enhancement of endotoxin-induced iNOS expression in atherosclerotic rabbits compared with normal New Zealand White rabbits.[180] In a chronic rejection model of transplant atherosclerosis in the rat, both macrophages and smooth muscle cells stained positive for iNOS.[181] More recently, it has been reported that iNOS is present within human atherosclerotic lesions and co-localizes with nitrotyrosine in macrophages and smooth muscle cells.[182] Induction of iNOS was also observed in the endothelium and smooth muscle of intramyocardial vessels of patients with ischaemic heart disease.[183]

The observation that iNOS induction in vascular smooth muscle cells, as in macrophages, is accompanied by upregulation of L-arginine transport,[184] may contribute to the stimulating effect of L-arginine on vessel relaxation in some experimental settings.

### Mechanical injury and iNOS

The hypocontractility to several agonists observed after balloon denudation of rat[185,186] or balloon angioplasty of rabbit arteries[187] was also attributed to the induction of iNOS in the vessel wall and was already noticeable 6 h post-injury.[186] Unlike the case in normal arteries, L-arginine evoked significant relaxation in deendothelialized balloon-injured vessel segments, which was reversed by the NOS inhibitor L-NAME.[187] In the balloon-injured rat carotid artery, reverse transcription and polymerase chain reaction amplification showed the appearance of iNOS mRNA already 24 h post surgery, and *in situ* hybridization located iNOS mRNA in neointimal smooth muscle cells, particularly at the luminal side of the vessel, conferring a nonthrombogenic surface.[188]

Cytokines introduced in the affected vessel by infiltrating monocytes and T-lymphocytes may provide the stimulus for iNOS induction in the smooth muscle cells. Also, oxLDL[189] and LDL[190,191] have been shown to upregulate iNOS activity in macrophages and vascular smooth muscle cells under certain conditions. On the other hand, mediators that inhibit iNOS induction e.g. heat shock proteins[192] or NO itself,[193] may determine the final output of NO.

### NO: A Radical with Anti-atherogenic Properties

Since the impairment in the EDRF/NO pathway occurs early or even precedes the development of visible lesions in the process of atherosclerosis, many authors have speculated on a causal role of this functional defect. This view is supported by a number of *in vitro* studies demonstrating the suppression by NO, produced endogenously or derived from NO donors, of several key processes involved in atherogenesis (Table 1).

### In vitro studies

*Interference with oxidative processes*

Since superoxide anion contributes to oxidative stress, LDL modification[194] and inflammatory gene transcription via the activation of NF-κB,[13] the decreased formation or inactivation of superoxide by NO may be considered protective. In this respect, it has been shown that the NO derived from iNOS inhibits xanthine oxidase in interferon-γ-stimulated macrophages[195] and that authentic exogenous NO inhibits xanthine oxidase in a cell-free system,[196] possibly by reversible alteration of the flavin prosthetic site.[197] Nitric oxide also inhibits neutrophil superoxide anion production via a direct action on the NADPH oxidase.[196] The NO donors known as NONOates abrogate the cytotoxic effects of superoxide on Chinese hamster lung fibroblasts.[198] Moreover, NO also protected against cellular damage by other reactive oxygen species e.g. hydrogen peroxide and alkyl peroxides, by several mechanisms such as prevention of haem oxidation, inhibition of Fenton-type oxidation of DNA, and abatement of lipid

**Table 1**. Anti-atherogenic properties of nitric oxide in vitro

| | Reference |
|---|---|
| *Interference with oxidative processes* | |
| Cytoprotection against oxidative stress | 198–200 |
| Inhibition of cell-mediated LDL oxidation | 201–205, 210 |
| Inhibition of lipoxygenase activity | 207 |
| Inhibition of oxLDL cytotoxicity | 211 |
| Inactivation of xanthine oxidase | 195–197 |
| Inhibition of NADPH oxidase | 196 |
| Reduction of endothelial hyperpermeability | 212 |
| *Interference with leukocyte recruitment* | |
| Suppression endothelial adhesion molecules | 214, 217 |
| Inhibition of monocyte chemotaxis | 219 |
| Inhibition of monocyte adhesion | 217, 219, 220 |
| Inhibition of neutrophil adhesion | 60, 213–216 |
| Inhibition of MCP-1 expression | 218 |
| Inhibition of NF-κB activation | 217, 218 |
| *Antiproliferative actions* | |
| Inhibition of smooth muscle cell proliferation | 221–227 |
| Inhibition of smooth muscle cell migration | 228, 229 |
| Inhibition of T-cell proliferation | 230–232 |
| Stimulation of endothelial repair | 233 |
| *Inhibition of platelet activation* | 234–236 |

peroxidation.[199] Activation of the stress protein haem oxygenase by NO may contribute to its cytoprotective effect.[200] Furthermore, NO reduced the oxidative modification of LDL by macrophages,[201–204] endothelial cells[205] and lipoxygenase[206] by acting as a potent terminator of radical chain propagation reactions.[206] Also, as 15-lipoxygenase has been implicated in LDL oxidation,[8] NO might protect LDL by inhibiting lipoxygenase activity.[207] Conversely, the decreased expression of iNOS activity in oxLDL-laden foam cells[208,209] has been implicated in the accelerated oxidation of LDL by these macrophages.[210] Furthermore, NO released by donor compounds inhibited the cellular toxicity of the lipid hydroperoxides contained in oxLDL, presumably by scavenging the propagatory free radicals generated during peroxidation of the endothelial cell membranes.[211] NO donors also blocked the hydrogen peroxide-related increase in endothelial permeability by a cyclic GMP-mediated mechanism.[212]

*Interference with leukocyte recruitment*

Inhibition of NO synthase in endothelial cells by L-NAME increased the intracellular oxidative stress, resulting in enhanced adhesion of neutrophils via CD18/ICAM-1 interaction[213] or the upregulation of P-selectin on the endothelium.[214] Neutrophil adhesion to the endothelium was augmented by hypercholesterolaemia[60,215] and this increase was prevented by the NO donor SPM 5185.[216] Inhibition of NO biosynthesis also induced the expression of VCAM-1[217] and upregulated MCP-1 mRNA and protein in cultured human endothelial cells, whereas addition of the NO donor SIN-1 dose-dependently decreased MCP-1 mRNA expression and secretion, presumably by suppressing a NF-κB-like transcriptional regulator.[218] Authentic NO gas inhibited monocyte adhesion and chemotaxis[219] and exposure to shear stress inhibited monocyte adhesion by an NO-dependent mechanism.[220] Furthermore, NO donors decreased the cytokine-induced expression of the endothelial adhesion molecules VCAM-1, ICAM-1 and E-selectin.[217]

*Antiproliferative action of nitric oxide*

As the proliferation of vascular smooth muscle cells, macrophages and T-lymphocytes contributes to the progression of intimal lesions, cell growth inhibition by NO could significantly reduce lesion formation. Nitric oxide has been shown by several investigators to inhibit smooth muscle cell proliferation[221–227] as well as migration[228,229] *in vitro*. Both effects were cyclic GMP-mediated. T-cell proliferation is also reduced by NO.[230–232]

Interestingly, the effect of NO appeared to be quite different in endothelial cells, in that it induced endothelial cell growth and motility *in vitro* and mediated the mitogenic effect of vascular endothelial growth factor.[233]

*Antiplatelet effects of nitric oxide*

Although the inhibitory effect of NO on platelet adhesion and aggregation[234,235] (reviewed by Bassenge[236]) is often considered an anti-atherogenic effect of NO, platelets are minimally involved during the early stages of the atherogenic process. However, the endothelial surface over advanced human plaques often shows focal loss of cells[15] and platelet adhesion may promote the progression of those lesions, eventually leading to plaque fissuring and thrombosis. Also following gross mechanical injury evoked by balloon angioplasty, platelet-derived products have been proposed to contribute to neointima formation.[4]

### Inhibition of atherosclerosis by NO in vivo

Several *in vivo* studies (Table 2) support the concept that NO may suppress both atherosclerosis and intimal thickening. Oral L-arginine

**Table 2.** Anti-atherogenic properties of nitric oxide in vivo

| | Reference |
|---|---|
| *Modulation of leukocyte-endothelial interaction* | |
| attenuation by L-arginine | 238 |
| attenuation by NO donors | 240, 241 |
| increase by NOS inhibitors | 239 |
| *Modulation of fatty streak formation in cholesterol-fed rabbits* | |
| *reduction by L-arginine* | 123, 237 |
| reduction by NO donor | 242 |
| increase by NOS inhibitors | 243, 244 |
| *Modulation of intimal thickening evoked by different stimuli* | |
| *balloon denudation* | |
| reduction by L-arginine | 134, 250, 251 |
| reduction by EDRF/NO | 252–254 |
| reduction by eNOS transfection | 259 |
| reduction by exogenous NO | 255, 256, 258 |
| increase by NOS inhibitors | 134, 250, 251 |
| balloon angioplasty | |
| reduction by L-arginine | 134 |
| collar | |
| reduction by NO donors | 260 |
| vein graft | |
| reduction by L-arginine | 249 |

supplementation caused a striking inhibition of fatty streak formation in hypercholesterolaemic rabbits.[237] Since the adhesion of monocytes to endothelial cells is imperative to lesion formation in this model, the authors further addressed the effects of the NO precursor on the adhesion of a murine monocytic cell line to the aortic endothelium of cholesterol-fed rabbits *ex vivo*.[238] The enhanced endothelial adhesiveness for monocytes in hypercholesterolaemic aortas was significantly reduced if the rabbits had received supplemental dietary arginine and significantly increased in rabbits treated with L-NAME. This was associated with, respectively, increased or decreased elaboration of vascular nitrogen oxides as measured by chemiluminescence. The observation that hypercholesterolaemia-induced impairment in endothelium-dependent relaxation was only marginally improved by L-arginine treatment while lesion formation was significantly reduced,[123] suggests that the beneficial effect of L-arginine may primarily infer from the increased activity of iNOS. However, it should be noted again that arginine and other basic amino acids are potent hormonal secretagogues in adrenals and many other endocrine organs.[133] Hence, part of the anti-atherosclerotic effect of systemic arginine administration might be related to the release of glucocorticoids or other immunosuppressive hormones which are known to suppress intimal thickening and experimental atherosclerosis.

The ambiguity of results obtained with L-arginine is avoided in studies with NO donor compounds or NOS inhibitors. L-NMMA and L-NAME increased leukocyte adhesion *in vivo* by a CD11/CD18-dependent mechanism.[239] Conversely, the NO donor SIN-1 prevented leukocyte adhesion. The observation that both SOD and SIN-1 inhibited leukocyte adhesion only under conditions associated with superoxide formation suggests that the anti-adhesive properties of NO may relate to its ability to inactivate the superoxide anion.[240] Another NO donor attenuated leukocyte endothelial interaction in an *in vivo* model of ischaemia-reperfusion, and this appeared to be in part mediated through a decreased expression of endothelial P-selectin.[241] Pentaerythrityl tetranitrate, an organic nitrate, has been documented to inhibit cholesterol-induced fatty streak formation in rabbits, but the beneficial effect was not seen with isosorbide mononitrate, another organic nitrate.[242] This could be due to differences with respect to the development of tolerance or the NO releasing capacity between the two organic nitrates. Conversely, treatment with molsidomine, whose active metabolite is the spontaneous NO donor SIN-1, actually enhanced lesion formation in the hypercholesterolaemic rabbit.[125] This may relate to the generation of superoxide anion from SIN-1, which could abrogate the beneficial effects of the simultaneously released NO.

Moreover, oral or parenteral treatment with the NO synthase inhibitor L-NAME for 4 to 12 weeks[243,244] enhanced fatty streak formation significantly. Therefore, the data suggest that vascular NO, produced by eNOS or iNOS, inhibits *de novo* formation of intimal lesions. However, this conclusion is somewhat confounded by the observation that L-NAME augmented plasma cholesterol levels in these rabbits, particularly after prolonged treatment. Since hypercholesterolaemia is the ultimate driving force for the lesions in this model, it is conceivable that this contributed to the accelerated atherosclerosis.

The inhibitory effect of NO on atherosclerosis may result from the above described *in vitro* actions, but NO-mediated decrease of endothelin production[28,245] may also be involved. Endothelin is a potent mitogen[246] and inducer of collagen synthesis[247] in vascular smooth muscle cell cultures, and its production may be increased in atherosclerosis.[248]

### Inhibition of intimal thickening by NO

The interferences with cholesterol absorption or metabolism are circumvented in studies of intimal thickening in animals with normal plasma cholesterol levels. Oral L-arginine supplementation suppressed intimal hyperplasia in experimental vein grafts[249] and after balloon denudation of the rat carotid artery.[250,251] The NOS inhibitor L-NAME reversed the effect of arginine,[250] indicating that the attenuation of the intimal hyperplasia was mediated by NO. The NO is presumably formed by iNOS,[185,186,188] since regrowth of the endothelial cells is virtually absent, and eNOS activity does not recover. Moreover, the endogenous biosynthesis of NO appears to modulate the process, since systemic[250] or local, perivascular[251] administration of L-NAME aggravated intimal thickening in response to balloon denudation. Increasing the flow in the injured carotid artery by ligating the contralateral artery significantly reduced intimal thickening, and this effect was in part mediated by endogenous NO.[252] Likewise, the protective effect of angiotensin converting enzyme (ACE) inhibitors, which also block kinin degradation, may be mediated in part by stimulation of the endogenous production of NO by bradykinin.[253,254]

Conversely, treatments with exogenous NO by oral administration of the cysteine-containing NO donor SPM-5185[255] or by chronic inhalation of NO[256] were effective in reducing the size of intimal lesions after injury of the rat carotid artery. Nitroglycerin treatment only decreased the initial medial smooth muscle cell proliferation without affecting the thickness of the neointima after 3 weeks.[257] This may be due to insufficient NO formation as a result of the well known development of tolerance associated with this class of nitrovasodilators.[101] A single local treatment of the denuded rabbit femoral artery with a protein adduct of NO inhibited platelet deposition and neointimal proliferation in the injured rabbit femoral artery.[258] *In vivo* eNOS gene transfer in the vessel wall after denudation of the rat carotid artery provided further evidence for the inhibition of smooth muscle cell accumulation by NO. Transfection of the eNOS gene in the media not only restored the calcium-dependent NO production and concomitant relaxations of the denuded artery, it also inhibited neointima formation at day 14 after balloon injury by 70%.[259] This experiment provides direct evidence that NO is an endogenous inhibitor of vascular lesion formation *in vivo*. Furthermore, these experiments suggest the possibility of eNOS transfection or local delivery of long-lived NO adducts as potential therapeutic approaches to treat neointimal hyperplasia.

The inhibition of intimal thickening by NO is not restricted to models characterized by endothelial denudation, but is also seen when intimal thickening is induced in rabbit arteries by the perivascular placement of a collar. Oral treatment with the NO-donor SPM-5185 reduced the collar-induced intimal thickening, whereas only a tendency towards inhibition was observed by treatment with molsidomine, whose active metabolite is the NO donor SIN-1.[260] It is not clear whether the difference between the two drugs was related to the dose, or different characteristics of the NO donors, i.e. the presence of sulphydryl groups in SPM-5185 or the release of superoxide anion from SIN-1.

Finally, there are indications that NO inhibits neointima formation induced by balloon angioplasty of lesion-free animal arteries. The vessel wall distension by the balloon creates a much more extensive injury of the media, predisposition to thrombus formation and accelerated intimal thickening. Although the endothelial cells regenerate quickly, vascular reactivity studies show that the eNOS pathway remains dysfunctional,[134,261] whereas iNOS is induced in non-endothelial vascular cells.[187] Oral L-arginine supplementation improved the endothelium-dependent vasorelaxation and suppressed the intimal hyperplasia after balloon angioplasty of rabbit iliac arteries.[134] In accordance with the finding in the collar model, treatment with the NO donor SIN-1 did not influence intimal thickening following porcine carotid angioplasty, although the compound was effective in inhibiting medial smooth muscle cell proliferation.[262]

Also in two other models of intimal thickening a clear relationship between inhibition of smooth muscle cell mitosis and neointima formation is lacking. Smooth muscle cell mitosis was influenced less than intimal thickening after eNOS gene transfer in denuded rat arteries[259] and after NO donor treatment of rabbit collared arteries.[260] This suggests that NO exerts its major effect on smooth muscle cell migration,[228,229] which is a crucial event in intimal thickening. Whether inhibition of migration is of importance to human atherosclerosis remains to be determined, as atherosclerosis develops in an existing intima[1,3] and migration of smooth muscle cells from media to intima is not considered a major determinant in atherogenesis.[4]

### NO: A Radical Promoter of Atherosclerosis (Table 3)

### LDL oxidation

Nitric oxide is a nitrogen-free radical and can initiate lipid peroxidation in LDL in the absence[204,263] or presence[201,264–266] of superoxide anion. In the former case, LDL modification appeared to be restricted to an increase in lipid hydroperoxide content[201] without further evolution to a high-uptake form recognized by the scavenger receptor.[264] Nitrite- and NO-oxidized LDL demonstrated the biological properties of minimally oxidized LDL.[263] More extensive LDL oxidation occurred if superoxide anion was present e.g. during the decomposition of SIN-1.[264] Superoxide combines with nitric oxide to form the stronger oxidants peroxynitrite and its decomposition product the hydroxyl radical.[267–269]

**Table 3.** Pro-atherogenic properties of NO

| | Reference |
|---|---|
| Oxidation of LDL | 201, 204, 263–267 |
| Cytotoxic effects | 20, 268, 272–274 |
| Induction of apoptosis | 276, 277, 286 |
| Increased matrix breakdown | 282–284 |

### Cytotoxic effects

The concept that peroxynitrite formation occurs in atherosclerosis is strongly supported by the immunohistochemical demonstration of extensive nitration of protein tyrosines in advanced human lesions.[270] The presence of 3-nitro-L-tyrosine, quantified in the human brain by high-performance liquid chromatography, is also considered indicative of oxidative stress induced by reactive oxygen intermediates and nitric oxide.[271] Excessive NO synthesis and peroxynitrite formation have been implicated in cytotoxic effects in endothelial cells,[268,272] smooth muscle cells[273] and macrophages.[274] Cell damage results from the inhibition of mitochondrial respiration, aconitase activity and DNA synthesis, as well as from iron loss.[20] On the other hand, nitric oxide-induced p53 accumulation safeguarded against DNA damage through p53-mediated suppression of iNOS gene expression, thus reducing the potential for NO-induced DNA damage.[275] The release of basic fibroblast growth factor from damaged vascular smooth muscle cells may counteract the toxic effects on the endothelium by stimulating endothelial cell proliferation.[273] In view of these findings, the protective effects of antioxidants in several models of atherosclerosis may in part derive from the prevention of NO breakdown by oxygen radicals.

### Induction of apoptosis

Nitric oxide has also been reported to cause apoptosis or programmed cell death in macrophages[276,277] and smooth muscle cells.[278] Apoptosis participates in the regulation of the cellularity of intimal lesions in balloon-injured arteries[279] and human atherosclerosis.[280] Theoretically, augmentation of apoptosis by NO could retard plaque growth, which may be considered beneficial. However, an imbalance between proliferation and apoptosis has been suggested to underlie the development of the cell-poor, necrotic core.[281] The size of the core determines the stability of the plaque. Stimulation of apoptotic cell death by NO or other molecules may thus increase the risk of plaque fissure and thromboembolic complications.

### Matrix breakdown

Enhanced matrix breakdown by the activation of matrix metalloproteinases by NO[282,283] or the inactivation of the tissue inhibitor of metalloproteinase-1 by peroxynitrite[284] may contribute to the destabilization of the lesions and may promote the development of a necrotic core in advanced plaques.

In summary, it has been known for a decade that the loss of endothelial NO production impairs endothelium-dependent dilatation and promotes vasospasm in atherosclerotic arteries. More recent evidence indicates that dysfunction of the endothelial NO pathway may promote atherosclerosis in view of the described protective effects of NO against leukocyte adhesion, oxidative processes, smooth muscle cell migration and proliferation. On the other hand, there is ample evidence to consider NO as a molecular aggressor in chronic inflammatory processes like atherosclerosis.[285]

### References

1. Stary HC, Blankenhorn DH, Chandler AB, Glagov S, Insull W Jr, Richardson M, Rosenfeld ME, Schaffer SA, Schwartz CJ, Wagner WD, Wissler RW. A definition of the intima of human arteries and of its atherosclerosis-prone regions. *Circulation* 1992; **85**: 391–405.
2. Thubrikar MJ, Robicsek F. Pressure-induced arterial wall stress and atherosclerosis. *Ann Thorac Surg* 1995; **59**: 1594–1603.
3. Davies MJ, Woolf N. Atherosclerosis: what is it and why does it occur? *Br Heart J* 1993; **69 (suppl)**: S3–S11.
4. Jackson CL. Animal models of restenosis. *Trends Cardiovasc Med* 1994; **4**: 122–130.
5. Kockx MM, De Meyer GRY, Jacob WA, Bult H, Herman AG. Triphasic sequence of neointimal formation in the cuffed carotid artery of the rabbit. *Arterioscler Thromb* 1992; **12**: 1447–1457.
6. Ross R. The pathogenesis of atherosclerosis: a perspective for the 1990s. *Nature* 1993; **362**: 801–809.
7. Libby P, Hansson GK. Biology of disease. Involvement of the immune system in human atherogenesis: current knowledge and unanswered questions. *Lab Invest* 1991; **64**: 5–15.
8. Witztum JL. Role of oxidised low density lipoprotein in atherogenesis. *Br Heart J* 1993; **69 (suppl)**: S12–S18.
9. Holvoet P, Collen D. Oxidized lipoproteins in atherosclerosis and thrombosis. *FASEB J* 1994; **8**: 1279–1284.
10. Thomas JP, Geiger PG, Girotti AW. Lethal damage to endothelial cells by oxidized low density lipoprotein: role of selenoperoxidases in cytoprotection against lipid hydroperoxide- and iron-mediated reactions. *J Lipid Res* 1993; **34**: 479–490.
11. Kume N, Cybulsky MI, Gimbrone MA Jr. Lysophosphatidylcholine, a component of atherogenic lipoproteins, induces mononuclear leukocyte adhesion molecules in cultured human and rabbit arterial endothelial cells. *J Clin Invest* 1992; **90**: 1138–1144.
12. Khan BV, Parthasarathy SS, Alexander RW, Medford RM. Modified low density lipoprotein and its constituents augment cytokine-activated vascular cell adhesion molecule-1 gene expression in human vascular endothelial cells. *J Clin Invest* 1995; **95**: 1262–1270.
13. Collins T. Biology of disease. Endothelial nuclear factor-kB and the initiation of the atherosclerotic lesion. *Lab Invest* 1993; **68**: 499–508.
14. Furchgott RF, Zawadzki JV. The obligatory role of endothelial cells in the relaxation of arterial smooth muscle by acetylcholine. *Nature* 1980; **288**: 373–376.
15. Ignarro LJ, Buga GM, Wood KS, Byrns RE, Chaudhuri G. Endothelium-derived relaxing factor produced and released from artery and vein is nitric oxide. *Proc Natl Acad Sci USA* 1987; **84**: 9265–9269.
16. Palmer RM, Ferrige AG, Moncada S. Nitric oxide release accounts for the biological activity of endothelium-derived relaxing factor. *Nature* 1987; **327**: 524–526.
17. Feelisch M, te Poel M, Zamora R, Deussen A, Moncada S. Understanding the controversy over the identity of EDRF. *Nature* 1994; **368**: 62–65.
18. Myers PR, Minor RL Jr, Guerra R Jr, Bates JN, Harrison DG. Vasorelaxant properties of the endothelium-derived relaxing factor more closely resemble S-nitrosocysteine than nitric oxide. *Nature* 1990; **345**: 161–163.
19. Rubanyi GM, Johns A, Wilcox D, Bates FN, Harrison D. Evidence that a S-nitrosothiol, but not nitric oxide, may be identical with endothelium-derived relaxing factor. *J Cardiovasc Pharmacol* 1991; **17 (suppl 3)**: S41–S45.
20. Gross SS, Wolin MS. Nitric oxide: pathophysiological mechanisms.

*Annu Rev Physiol* 1995; **57**: 737–769.
21. Busse R, Mülsch A, Fleming I, Hecker M. Mechanisms of nitric oxide release from the vascular endothelium. *Circulation* 1993; **87 (suppl V)**: V-18–V-25.
22. Martin GR. Vascular receptors for 5-hydroxytryptamine: distribution, function and classification. *Pharmac Ther* 1994; **62**: 283–324.
23. Warner TD, Mitchell JA, Sheng H, Murad F. Effects of cyclic GMP on smooth muscle relaxation. *Adv Pharmacol* 1994; **26**: 171–194.
24. Murad F. Regulation of cytosolic guanylyl cyclase by nitric oxide: the NO-cyclic GMP signal transduction system. *Adv Pharmacol* 1994; **26**: 19–33.
25. Rapoport RM, Draznin MB, Murad F. Endothelium-dependent relaxation in rat aorta may be mediated through cyclic GMP-dependent protein phosphorylation. *Nature* 1996; **306**: 174–176.
26. Boulanger C, Lüscher TF. Release of endothelin from the porcine aorta. Inhibition by endothelium-derived nitric oxide. *J Clin Invest* 1990; **85**: 587–590.
27. Yanagisawa M, Kurihara H, Kimura S, Tomobe Y, Kobayashi M, Mitsui Y, Yazaki Y, Goto K, Masaki T. A novel potent vasoconstrictor peptide produced by vascular endothelial cells. *Nature* 1988; **332**: 411–415.
28. Brunner F, Stessel H, Kukovetz WR. Novel guanylyl cyclase inhibitor, ODQ reveals role of nitric oxide, but not of cyclic GMP in endothelin-1 secretion. *FEBS Lett* 1995; **376**: 262–266.
29. Schroeder JS, Bolen JL, Quint RA, Clark DA, Hayden WG, Higgins, CB, Wexler L. Provocation of coronary spasm with ergonovine maleate. New test with results in 57 patients undergoing coronary arteriography. *Am J Cardiol* 1977; **40**: 487–491.
30. Shimokawa H, Tomoike H, Nabeyama S, Yamamoto H, Araki H, Nakamura M. Coronary artery spasm induced in atherosclerotic miniature swine. *Science* 1983; **221**: 560–561.
31. Ginsburg R, Bristow MR, Davis K, Dibiase A, Billingham ME. Quantitative pharmacologic responses of normal and atherosclerotic isolated human epicardial coronary arteries. *Circulation* 1984; **69**: 430–440.
32. Kawachi Y, Tomoike H, Maruoka Y, Kikuchi Y, Araki H, Ishii Y, Tanaka K, Nakamura M. Selective hypercontraction caused by ergonovine in the canine coronary artery under conditions of induced atherosclerosis. *Circulation* 1984; **69**: 441–450.
33. Yokoyama M, Akita H, Mizutani T, Fukuzaki H, Watanabe Y. Hyperreactivity of coronary arterial smooth muscles in response to ergonovine from rabbits with hereditary hyperlipidemia. *Circ Res* 1983; **53**: 63–71.
34. Henry PD, Yokoyama M. Supersensitivity of atherosclerotic rabbit aorta to ergonovine. *J Clin Invest* 1980; **66**: 306–313.
35. Verbeuren TJ, Jordaens FH, Zonnekeyn LL, Van Hove CE, Coene M-C, Herman AG. Effect of hypercholesterolemia on vascular reactivity in the rabbit. I. Endothelium-dependent and endothelium-independent contractions and relaxations in isolated arteries of control and hypercholesterolemic rabbits. *Circ Res* 1986; **58**: 552–564.
36. Cohen RA, Shepherd JT, Vanhoutte PM. Inhibitory role of the endothelium in the response of isolated coronary arteries to platelets. *Science* 1983; **221**: 273–274.
37. Cocks TM, Angus JA. Endothelium-dependent relaxation of coronary arteries by noradrenaline and serotonin. *Nature* 1983; **305**: 627–630.
38. Heistad DD, Armstrong ML, Marcus ML, Piegors DJ, Mark AL. Augmented responses to vasoconstrictor stimuli in hypercholesterolemic and atherosclerotic monkeys. *Circ Res* 1984; **54**: 711–718.
39. Freiman PC, Mitchell GG, Heistad DD, Armstrong ML, Harrison DG. Atherosclerosis impairs endothelium-dependent vascular relaxation to acetylcholine and thrombin in primates. *Circ Res* 1986; **58**: 783–789.
40. Jayakody RL, Senaratne MP, Thomson AB, Kappagoda CT. Cholesterol feeding impairs endothelium-dependent relaxation of rabbit aorta. *Can J Physiol Pharmacol* 1985; **63**: 1206–1209.
41. Sreeharan N, Jayakody RL, Senaratne MP, Thomson AB, Kappagoda CT. Endothelium-dependent relaxation and experimental atherosclerosis in the rabbit aorta. *Can J Physiol Pharmacol* 1986; **64**: 1451–1453.
42. Habib JB, Bossaller C, Wells S, Williams C, Morrisett JD, Henry PD. Preservation of endothelium-dependent vascular relaxation in cholesterol-fed rabbit by treatment with the calcium blocker PN 200110. *Circ Res* 1986; **58**: 305–309.
43. Bossaller C, Habib GB, Yamamoto H, Williams C, Wells S, Henry PD. Impaired muscarinic endothelium-dependent relaxation and cyclic guanosine 5′-monophosphate formation in atherosclerotic human coronary artery and rabbit aorta. *J Clin Invest* 1987; **79**: 170–174.
44. Tagawa H, Tomoike H, Nakamura M. Putative mechanisms of the impairment of endothelium-dependent relaxation of the aorta with atheromatous plaque in heritable hyperlipidemic rabbits. *Circ Res* 1991; **68**: 330–337.
45. Jayakody L, Senaratne M, Thomson A, Kappagoda T. Endothelium-dependent relaxation in experimental atherosclerosis in the rabbit. *Circ Res* 1987; **60**: 251–264.
46. Galle J, Busse R, Bassenge E. Hypercholesterolemia and atherosclerosis change vascular reactivity in rabbits by different mechanisms. *Arterioscler Thromb* 1991; **11**: 1712–1718.
47. Chappell SP, Lewis MJ, Henderson AH. Effect of lipid feeding on endothelium dependent relaxation in rabbit aortic preparations. *Cardiovasc Res* 1987; **21**: 34–38.
48. Kolodgie FD, Virmani R, Rice HE, Mergner WJ. Vascular reactivity during the progression of atherosclerotic plaque. A study in Watanabe heritable hyperlipidemic rabbits. *Circ Res* 1990; **66**: 1112–1126.
49. Kanamura K, Waga S, Tochio H, Nagatani K. The effect of atherosclerosis on endothelium-dependent relaxation in the aorta and intracranial arteries of rabbits. *J Neurosurg* 1989; **70**: 793–798.
50. Shimokawa H, Vanhoutte PM. Impaired endothelium-dependent relaxation to aggregating platelets and related vasoactive substances in porcine coronary arteries in hypercholesterolemia and atherosclerosis. *Circ Res* 1989; **64**: 900–914.
51. Cohen RA, Zitnay KM, Haudenschild CC, Cunningham LD. Loss of selective endothelial cell vasoactive functions caused by hypercholesterolemia in pig coronary arteries. *Circ Res* 1988; **63**: 903–910.
52. Yamamoto Y, Tomoike H, Egashira K, Nakamura M. Attenuation of endothelium-related relaxation and enhanced responsiveness of vascular smooth muscle to histamine in spastic coronary arterial segments from miniature pigs. *Circ Res* 1987; **61**: 772–778.
53. Komori K, Shimokawa H, Vanhoutte PM. Hypercholesterolemia impairs endothelium-dependent relaxations to aggregating platelets in porcine iliac arteries. *J Vasc Surg* 1989; **10**: 318–325.
54. Yu SM, Huang ZS, Wang CY, Teng CM. Effects of hyperlipidemia on the vascular reactivity in the Wistar-Kyoto and spontaneously hypertensive rats. *Eur J Pharmacol* 1993; **248**: 289–295.
55. Harrison DG, Freiman PC, Armstrong ML, Marcus ML, Heistad DD. Alterations of vascular reactivity in atherosclerosis. *Circ Res* 1987; **61 (suppl II)**: II-74–II-80.
56. Förstermann U, Mügge A, Alheid U, Haverich A, Frölich JC. Selective attenuation of endothelium-mediated vasodilation in atherosclerotic human coronary arteries. *Circ Res* 1988; **62**: 185–190.
57. Berkenboom G, Depierreux M, Fontaine J. The influence of atherosclerosis on the mechanical responses of human isolated coronary arteries to substance P, isoprenaline and noradrenaline. *Br J Pharmacol* 1987; **92**: 113–120.
58. Chester AH, O'Neil GS, Moncada S, Tadjkarimi S, Yacoub MH. Low basal and stimulated release of nitric oxide in atherosclerotic epicardial coronary arteries. *Lancet* 1990; **336**: 897–900.
59. Verbeuren TJ, Simonet S, Vallez M-O, Laubie A. 5-$HT_2$-receptor blockade restores vasodilations to 5-HT in atherosclerotic rabbit hearts: role of the endothelium. *J Cardiovasc Pharmacol* 1991; **17 (suppl 3)**: S222–S228.
60. Lefer AM, Ma X-L. Decreased basal nitric oxide release in hypercholesterolemia increases neutrophil adherence to rabbit coronary artery endothelium. *Arterioscler Thromb* 1993; **13**: 771–776.
61. Böger RH, Bode-Böger SM, Gerecke U, Frölich JC. Long-term administration of L-arginine, L-NAME, and the exogenous NO donor molsidomine modulates urinary nitrate and cGMP excretion in rats. *Cardiovasc Res* 1994; **28**: 494–499.
62. Suzuki H, Ikenaga H, Hishikawa K, Nakaki T, Kato R, Saruta T. Increases in NO2-/NO3- excretion in the urine as an indicator of the release of endothelium-derived relaxing factor during elevation of blood pressure. *Clin Sci* 1992; **826**: 631–634.
63. Böger RH, Bode-Böger SM, Mügge A, Kienke S, Brandes R, Dwenger A, Frölich JC. Supplementation of hypercholesterolaemic rabbits with L-arginine reduces the vascular release of superoxide anions and restores NO production. *Atherosclerosis* 1995; **117**: 273–284.
64. Newman CM, Maseri A, Hackett DR, el-Tamimi HM, Davies GJ. Response of angiographically normal and atherosclerotic left anterior descending coronary arteries to acetylcholine. *Am J Cardiol* 1990; **66**: 1070–1076.
65. Bossaller C, Hehlert-Friedrich C, Jost S, Rafflenbeul W, Lichtlen P. Angiographic assessment of human coronary artery endothelial function by measurement of endothelium-dependent vasodilation. *Eur Heart J* 1989; **10 (suppl F)**: 44–48.
66. Gordon JB, Ganz P, Nabel EG, Fish RD, Zebede J, Mudge GH, Alexander RW, Selwyn AP. Atherosclerosis influences the vasomotor response of epicardial coronary arteries to exercise. *J Clin Invest* 1989; **83**: 1946–1952.
67. Horio Y, Yasue H, Rokutanda M, Nakamura N, Ogawa H, Takaoka K, Matsuyama K, Kimura T. Effects of intracoronary injection of acetylcholine on coronary arterial diameter. *Am J Cardiol* 1986; **57**: 984–989.
68. Ludmer PL, Selwyn AP, Shook TL, Wayne RR, Mudge GH, Alexander RW, Ganz P. Paradoxical vasoconstriction induced by acetylcholine in atherosclerotic coronary arteries. *New Engl J Med* 1986; **315**: 1046–1051.
69. Vrints CJM, Bult H, Bosmans J, Herman AG, Snoeck JP. Paradoxical vasoconstriction as result of acetylcholine and serotonin in diseased human coronary arteries. *Eur Heart J* 1992; **13**: 824–831.
70. Nabel EG, Selwyn AP, Ganz P. Large coronary arteries in humans are responsive to changing blood flow: an endothelium-dependent mechanism that fails in patients with atherosclerosis. *J Am Coll Cardiol* 1990; **16**: 349–356.
71. Drexler H, Zeiher AM, Wollschläger H, Meinertz T, Just H, Bonzel T. Flow-dependent coronary artery dilatation in humans. *Circulation* 1989; **80**: 466–474.

72. Cox DA, Vita JA, Treasure CB, Fish RD, Alexander RW, Ganz P, Selwyn AP. Atherosclerosis impairs flow-mediated dilation of coronary arteries in humans. *Circulation* 1989; **80**: 458–465.
73. McLenachan JM, Vita J, Fish RD, Treasure CB, Cox DA, Ganz P, Selwyn AP. Early evidence of endothelial vasodilator dysfunction at coronary branch points. *Circulation* 1990; **82**: 1169–1173.
74. Kawashima T, Yashiro A, Nandate H, Himeno E, Oka Y, Kaku T, Nakashima Y, Kuroiwa A. Increased susceptibility of angiographically smooth left anterior descending coronary artery to an impairment of vasoresponse to acetylcholine, and the relation between impaired vasoresponse and low-density lipoprotein cholesterol level. *Am J Cardiol* 1995; **75**: 1265–1267.
75. Vita JA, Treasure CB, Nabel EG, McLenachan JM, Fish RD, Yeung AC, Vekshtein VI, Selwyn AP, Ganz P. Coronary vasomotor response to acetylcholine relates to risk factors for coronary artery disease. *Circulation* 1990; **81**: 491–497.
76. Zeiher AM, Drexler H, Wollschläger H, Just H. Modulation of coronary vasomotor tone in humans. Progressive endothelial dysfunction with different early stages of coronary atherosclerosis. *Circulation* 1991; **83**: 391–401.
77. Vrints CJM, Bult H, Hitter E, Herman AG, Snoeck JP. Impaired endothelium-dependent cholinergic coronary vasodilation in patients with angina and normal coronary arteriograms. *J Am Coll Cardiol* 1992; **19**: 21–31.
78. Werns SW, Walton JA, Hsia HH, Nabel EG, Sanz ML, Pitt B. Evidence of endothelial dysfunction in angiographically normal coronary arteries of patients with coronary artery disease. *Circulation* 1989; **79**: 287–291.
79. Yasue H, Matsuyama K, Matsuyama K, Okumura K, Morikami Y, Ogawa H. Responses of angiographically normal human coronary arteries to intracoronary injection of acetylcholine by age and segment. Possible role of early coronary atherosclerosis. *Circulation* 1990; **81**: 482–490.
80. Rasheed Q, Hodgson JM. Application of intracoronary ultrasonography in the study of coronary artery pathophysiology. *J Clin Ultrasound* 1993; **21**: 569–578.
81. Vanhoutte PM, Shimokawa H. Endothelium-derived relaxing factor and coronary vasospasm. *Circulation* 1989; **80**: 1–9.
82. Meredith IT, Yeung AC, Weidinger FF, Anderson TJ, Uehata A, Ryan TJ Jr, Selwyn AP, Ganz P. Role of impaired endothelium-dependent vasodilation in ischemic manifestations of coronary artery disease. *Circulation* 1993; **87 (suppl V)**: V-56–V-66.
83. Stewart DJ, Münzel T, Bassenge E. Reversal of acetylcholine-induced coronary resistance vessel dilation by hemoglobin. *Eur J Pharmacol* 1987; **136**: 239–242.
84. Lüscher TF, Dohi Y, Tschudi M. Endothelium-dependent regulation of resistance arteries: alterations with aging and hypertension. *J Cardiovasc Pharmacol* 1992; **19 (suppl 5)**: S34–S42.
85. Rees DD, Palmer RMJ, Moncada S. Role of endothelium-derived nitric oxide in the regulation of blood pressure. *Proc Natl Acad Sci USA* 1989; **86**: 3375–3378.
86. Anderson TJ, Gerhard MD, Meredith IT, Charbonneau F, Delagrange D, Creager MA, Selwyn AP, Ganz P. Systemic nature of endothelial dysfunction in atherosclerosis. *Am J Cardiol* 1995; **75**: 71B–74B.
87. Sellke FW, Armstrong ML, Harrison DG. Endothelium-dependent vascular relaxation is abnormal in the coronary microcirculation of atherosclerotic primates. *Circulation* 1990; **81**: 1586–1593.
88. Kuo L, Davis MJ, Cannon MS, Chilian WM. Pathophysiological consequences of atherosclerosis extend into the coronary microcirculation. Restoration of endothelium-dependent responses by L-arginine. *Circ Res* 1992; **70**: 465–476.
89. Osborne JA, Siegman MJ, Sedar AW, Mooers SU, Lefer AM. Lack of endothelium-dependent relaxation in coronary resistance arteries of cholesterol-fed rabbits. *Am J Physiol* 1989; **256**: C591–C597.
90. Creager MA, Cooke JP, Mendelsohn ME, Gallagher SJ, Coleman SM, Loscalzo J, Dzau VJ. Impaired vasodilation of forearm resistance vessels in hypercholesterolemic humans. *J Clin Invest* 1990; **86**: 228–234.
91. Casino PR, Kilcoyne CM, Cannon RO, Quyyumi AA, Panza JA. Impaired endothelium-dependent vascular relaxation in patients with hypercholesterolemia extends beyond the muscarinic receptor. *Am J Cardiol* 1995; **75**: 40–44.
92. Yamamoto H, Bossaller C, Cartwright J Jr, Henry PD. Videomicroscopic demonstration of defective cholinergic arteriolar vasodilation in atherosclerotic rabbit. *J Clin Invest* 1988; **81**: 1752–1758.
93. Liao JK, Bettmann MA, Sandor T, Tucker JI, Coleman SM, Creager MA. Differential impairment of vasodilator responsiveness of peripheral resistance and conduit vessels in humans with atherosclerosis. *Circ Res* 1991; **68**: 1027–1034.
94. Egashira K, Inou T, Hirooka Y, Yamada A, Maruoka Y, Kai H, Sugimachi M, Suzuki S, Takeshita A. Impaired coronary blood flow response to acetylcholine in patients with coronary risk factors and proximal atherosclerotic lesions. *J Clin Invest* 1993; **91**: 29–37.
95. Celermajer DS, Sorensen KE, Gooch VM, Spiegelhalter DJ, Miller OI, Sullivan ID, Lloyd JK, Deanfield JE. Non-invasive detection of endothelial dysfunction in children and adults at risk of atherosclerosis. *Lancet* 1992; **340**: 1111–1115.
96. Uehata A, Gerhard MD, Meredith IT, Lieberman EL, Sylwyn AP, Creager M, Polak J, Ganz P, Yeung AC, Anderson TJ. Close relationship of endothelial dysfunction in coronary and brachial artery. *Circulation* 1993; **88**: I-618 (abstract).
97. Hayashi T, Fukuto JM, Ignarro LJ, Chaudhuri G. Basal release of nitric oxide from aortic rings is greater in female rabbits than in male rabbits: implications for atherosclerosis. *Proc Natl Acad Sci USA* 1992; **89**: 11259–11263.
98. Hashimoto M, Akishita M, Eto M, Ishikawa M, Kozaki K, Toba K, Sagara Y, Taketani Y, Orimo H, Ouchi Y. Modulation of endothelium-dependent flow-mediated dilatation of the brachial artery by sex and menstrual cycle. *Circulation* 1995; **92**: 3431–3435.
99. Flavahan NA. Atherosclerosis or lipoprotein-induced endothelial dysfunction. Potential mechanisms underlying reduction in EDRF/nitric oxide activity. *Circulation* 1992; **85**: 1927–1938.
100. Harrison DG, Ohara Y. Physiologic consequences of increased vascular oxidant stresses in hypercholesterolemia and atherosclerosis: implications for impaired vasomotion. *Am J Cardiol* 1995; **75**: 75B–81B.
101. Harrison DG, Bates JN. The nitrovasodilators. New ideas about old drugs. *Circulation* 1993; **87**: 1461–1467.
102. Verbeuren TJ, Jordaens FH, Van Hove CE, Van Hoydonck A-E, Herman AG. Release and vascular activity of endothelium-derived relaxing factor in atherosclerotic rabbit aorta. *Eur J Pharmacol* 1990; **191**: 173–184.
103. Minor RL Jr, Myers PR, Guerra R Jr, Bates JN, Harrison DG. Diet-induced atherosclerosis increases the release of nitrogen oxides from rabbit aorta. *J Clin Invest* 1990; **86**: 2109–2116.
104. Beetens JR, Coene M-C, Verheyen A, Zonnekeyn LL, Herman AG. Vitamin C increases the prostacyclin production and decreases the vascular lesions in experimental atherosclerosis in rabbits. *Prostaglandins* 1986; **32**: 335–352.
105. Beetens JR, Coene M-C, Verheyen A, Zonnekeyn LL, Herman AG. Biphasic response of intimal prostacyclin production during the development of experimental atherosclerosis. *Prostaglandins* 1986; **32**: 319–334.
106. Shimokawa H, Flavahan NA, Vanhoutte PM. Loss of endothelial pertussis toxin-sensitive G protein function in atherosclerotic porcine coronary arteries. *Circulation* 1991; **83**: 652–660.
107. Mangin EL Jr, Kugiyama K, Nguy JH, Kerns SA, Henry PD. Effects of lysolipids and oxidatively modified low density lipoprotein on endothelium-dependent relaxation of rabbit aorta. *Circ Res* 1993; **72**: 161–166.
108. Kugiyama K, Kerns SA, Morrisett JD, Roberts R, Henry PD. Impairment of endothelium-dependent arterial relaxation by lysolecithin in modified low-density lipoproteins. *Nature* 1990; **344**: 160–162.
109. Murohara T, Kugiyama K, Ohgushi M, Sugiyama S, Ohta Y, Yasue H. LPC in oxidized LDL elicits vasocontraction and inhibits endothelium-dependent relaxation. *Am J Physiol* 1994; **267**: H2441–H2449.
110. Inoue N, Hirata K-I, Yamada M, Hamamori Y, Matsuda Y, Akita H, Yokoyama M. Lyophosphatidylcholine inhibits bradykinin-induced phosphoinositide hydrolysis and calcium transients in cultured bovine aortic endothelial cells. *Circ Res* 1992; **71**: 1410–1421.
111. Flavahan NA. Lysophosphatidylcholine modifies G protein-dependent signaling in porcine endothelial cells. *Am J Physiol* 1993; **264**: H722–H727.
112. Kanazawa K, Kawashima S, Mikami S, Miwa Y, Hirata K-I, Suematsu M, Hayashi Y, Itoh H, Yokoyama M. Endothelial constitutive nitric oxide synthase protein and mRNA increased in rabbit atherosclerotic aorta despite impaired endothelium-dependent vascular relaxation. *Am J Pathol* 1996; **148**: 1949–1956.
113. Bialecki RA, Tulenko TN. Acute exposure to cholesterol increases arterial nitroprusside- and endothelium-mediated relaxation. *Am J Physiol* 1993; **264**: C32–C39.
114. Deliconstantinos G, Villiotou V, Stavrides JC. Modulation of particulate nitric oxide synthase activity and peroxynitrite synthesis in cholesterol enriched endothelial cell membranes. *Biochem Pharmacol* 1995; **49**: 1589–1600.
115. Hirata K-I, Miki N, Kuroda Y, Sakoda T, Kawashima S, Yokoyama M. Low concentration of oxidized low-density lipoprotein and lysophosphatidylcholine upregulate constitutive nitric oxide synthase mRNA expression in bovine aortic endothelial cells. *Circ Res* 1995; **76**: 958–962.
116. Liao JK, Shin WS, Lee WY, Clark SL. Oxidized low-density lipoprotein decreases the expression of endothelial nitric oxide synthase. *J Biol Chem* 1995; **270**: 319–324.
117. Pritchard KAJ, Groszek L, Smalley DM, Sessa WC, Wu M, Villalon P, Wolin MS, Stemerman MB. Native low-density lipoprotein increases endothelial cell nitric oxide synthase generation of superoxide anion. *Circ Res* 1995; **77**: 510–518.
118. Mügge A, Harrison DG. L-arginine does not restore endothelial dysfunction in atherosclerotic rabbit aorta in vitro. *Blood Vessels* 1991; **28**: 354–357.
119. Caparotta L, Pandolfo L, Chinellato A, Ragazzi E, Froldi G, Aliev G, Fassina G. L-arginin does not improve endothelium-dependent relaxation in in vitro Watanabe rabbit thoracic aorta. *Amino Acids* 1993; **5**: 403–411.

120. Bult H, Buyssens N, De Meyer GRY, Jordaens FH, Herman AG. Effects of chronic treatment with a source of exogenous nitric oxide on EDRF release by aortae from normal and hypercholesterolemic rabbits. In: Moncada S, Higgs EA, eds. *Nitric Oxide from L-arginine: A Bioregulatory System*. Amsterdam: Elsevier Science, 1990; 101–106.
121. White CR, Brock TA, Chang L-Y, Crapo J, Briscoe P, Ku D, Bradley WA, Gianturco SH, Gore J, Freeman BA, *et al*. Superoxide and peroxynitrite in atherosclerosis. *Proc Natl Acad Sci USA* 1994; **91**: 1044–1048.
122. Cooke JP, Andon NA, Girerd XJ, Hirsch AT, Creager MA. Arginine restores cholinergic relaxation of hypercholesterolemic rabbit thoracic aorta. *Circulation* 1991; **83**: 1057-1062.
123. Singer AH, Tsao PS, Wang B-Y, Bloch DA, Cooke JP. Discordant effects of dietary L-arginine on vascular structure and reactivity in hypercholesterolemic rabbits. *J Cardiovasc Pharmacol* 1995; **25**: 710–716.
124. Kaski JC, Crea F, Meran D, Rodriguez L, Araujo L, Chierchia S, Davies G, Maseri A. Local coronary supersensitivity to diverse vasoconstrictive stimuli in patients with variant angina. *Circulation* 1986; **74**: 1255–1265.
125. Bult H, De Meyer GRY, Herman AG. Influence of chronic treatment with a nitric oxide donor on fatty streak development and reactivity of the rabbit aorta. *Br J Pharmacol* 1995; **114**: 1371–1382.
126. Drexler H, Zeiher AM, Meinzer K, Just H. Correction of endothelial dysfunction in coronary microcirculation of hypercholesterolaemic patients by L-arginine. *Lancet* 1991; **338**: 1546–1550.
127. Bode-Böger SM, Böger RH, Alfke H, Heinzel D, Tsikas D, Creutzig A, Alexander K, Frölich JC. L-Arginine induces nitric oxide-dependent vasodilation in patients with critical limb ischemia. A randomized, controlled study. *Circulation* 1996; **93**: 85–90.
128. Trezzini C, Jungi TW, Spycher MO, Maly FE, Raos P. Human monocytes CD36 and CD16 are signaling molecules. Evidence from studies using antibody-induced chemiluminescence as a tool to probe signal transduction. *Immunology* 1990; **71**: 29–37.
129. Verbeuren TJ, Bonhomme E, Laubie M, Simonet S. Evidence for induction of non-endothelial NO-synthase in aortas of cholesterol-fed rabbits. *J Cardiovasc Pharmacol* 1993; **21**: 841–845.
130. Casino PR, Kilcoyne CM, Quyyumi AA, Hoeg JM, Panza JA. Investigation of decreased availability of nitric oxide precursor as the mechanism responsible for impaired endothelium-dependent vasodilation in hypercholesterolemic patients. *J Am Coll Cardiol* 1994; **23**: 844–850.
131. Girerd XJ, Hirsch AT, Cooke JP, Dzau VJ, Creager MA. L-arginine augments endothelium-dependent vasodilation in cholesterol-fed rabbits. *Circ Res* 1990; **67**: 1301–1308.
132. Creager MA, Gallagher SJ, Girerd XJ, Coleman SM, Dzau VJ, Cooke JP. L-Arginine improves endothelium-dependent vasodilation in hypercholesterolemic humans. *J Clin Invest* 1992; **90**: 1248–1253.
133. Barbul A. Physiology and pharmacology of arginine. In: Moncada S, Higgs EA, eds. *Nitric Oxide from L-arginine: A Bioregulatory System*. Amsterdam: Elsevier Science, 1990; 317–329.
134. Tarry WC, Makhoul RG. L-arginine improves endothelium-dependent vasorelaxation and reduces intimal hyperplasia after balloon angioplasty. *Arterioscler Thromb* 1994; **14**: 938–943.
135. Jeremy RW, McCarron H, Sullivan D. Effects of dietary L-arginine on atherosclerosis and endothelium-dependent vasodilatation in the hypercholesterolemic rabbit. Response according to treatment duration, anatomic site, and sex. *Circulation* 1996; **94**: 498–506.
136. Imaizumi T, Hirooka Y, Masaki H, Harada S, Momohara M, Tagawa T, Takeshita A. Effects of L-arginine on forearm vessels and responses to acetylcholine. *Hypertension* 1992; **20**: 511–517.
137. Bode-Böger SM, Böger RH, Creutzig A, Tsikas D, Gutzki FM, Alexander K, Frolich JC. L-arginine infusion decreases peripheral arterial resistance and inhibits platelet aggregation in healthy subjects. *Clin Sci* 1994; **87**: 303–310.
138. Kanno K, Hirata Y, Emori T, Ohta K, Eguchi S, Imai T, Marumo F. L-arginine infusion induces hypotension and diuresis/natriuresis with concomitant increased urinary excretion of nitrite/nitrate and cyclic GMP in humans. *Clin Exp Pharmacol Physiol* 1992; **19**: 619–625.
139. Vallance P, Leone A, Calver A, Collier J, Moncada S. Accumulation of an endogenous inhibitor of nitric oxide synthesis in chronic renal failure. *Lancet* 1992; **339**: 572–575.
140. Yu XJ, Li YJ, Xiong Y. Increase of an endogenous inhibitor of nitric oxide synthesis in serum of high cholesterol fed rabbits. *Life Sci* 1994; **54**: 753–758.
141. Xiong Y, Li YJ, Yu XJ, Liu GZ, Li NS. Endogenous inhibitors of nitric oxide synthesis and lipid peroxidation in hyperlipidemic rabbits. *Acta Pharmacol Sin* 1996; **17**: 149–152.
142. Buga GM, Griscavage JM, Rogers NE, Ignarro LJ. Negative feedback regulation of endothelial cell function by nitric oxide. *Circ Res* 1993; **73**: 808–812.
143. Griscavage JM, Fukuto JM, Komori Y, Ignarro LJ. Nitric oxide inhibits neuronal nitric oxide synthase by interacting with the heme prosthetic group. *J Biol Chem* 1994; **269**: 21644–21649.
144. Walter R, Schaffner A, Schoedon G. Differential regulation of constitutive and inducible nitric oxide production by inflammatory stimuli in murine endothelial cells. *Biochem Biophys Res Commun* 1994; **202**: 450–455.
145. Rubanyi GM, Vanhoutte PM. Superoxide anions and hyperoxia inactivate endothelium-derived relaxing factor. *Am J Physiol* 1986; **250**: H822–H827.
146. Gryglewski RJ, Palmer RMJ, Moncada S. Superoxide anion is involved in the breakdown of endothelium-derived vascular relaxing factor. *Nature* 1986; **320**: 454–456.
147. Rubanyi GM, Vanhoutte PM. Oxygen-derived free radicals, endothelium, and responsiveness of vascular smooth muscle. *Am J Physiol* 1986; **250**: H815–H821.
148. Abrahamsson T, Brandt U, Marklund SL, Sjöqvist P-O. Vascular bound recombinant extracellular superoxide dismutase type C protects against the detrimental effects of superoxide radicals on endothelium-dependent arterial relaxation. *Circ Res* 1992; **70**: 264–271.
149. Mügge A, Elwell JH, Peterson TE, Harrison DG. Release of intact endothelium-derived relaxing factor depends on endothelial superoxide dismutase activity. *Am J Physiol* 1991; **260**: C219–C225.
150. Ohara Y, Peterson TE, Harrison DG. Hypercholesterolemia increases endothelial superoxide anion production. *J Clin Invest* 1993; **91**: 2546–2551.
151. Mügge A, Brandes RP, Böger RH, Dwenger A, Bode-Böger S, Kienke S, Frölich JC, Lichtlen PR. Vascular release of superoxide radicals is enhanced in hypercholesterolemic rabbits. *J Cardiovasc Pharmacol* 1994; **24**: 994–998.
152. Chin JH, Azhar S, Hoffman BB. Inactivation of endothelial derived relaxing factor by oxidized lipoproteins. *J Clin Invest* 1992; **89**: 10–18.
153. Maeba R, Maruyama A, Tarutani O, Ueta N, Shimasaki H. Oxidized low-density lipoprotein induces the production of superoxide by neutrophils. *FEBS Lett* 1995; **377**: 309–312.
154. Ohara Y, Peterson TE, Zheng B, Kuo JF, Harrison DG. Lysophophatidylcholine increases vascular superoxide anion production via protein kinase C activation. *Arterioscler Thromb* 1994; **14**: 1007-1013.
155. Zulueta JJ, Yu FS, Hertig IA, Thannickal VJ, Hassoun PM. Release of hydrogen peroxide in response to hypoxia-reoxygenation: role of an NAD(P)H oxidase-like enzyme in endothelial cell plasma membrane. *Am J Respir Cell Mol Biol* 1995; **12**: 41–49.
156. Sharma P, Evans AT, Parker PJ, Evans FJ. NADPH-oxidase activation by protein kinase C-isotypes. *Biochem Biophys Res Commun* 1991; **177**: 1033–1040.
157. Henriksson P, Bergström K, Edhag O. Experimental atherosclerosis and a possible generation of free radicals. *Thromb Res* 1985; **38**: 195–198.
158. Sharma RC, Crawford DW, Kramsch DM, Sevanian A, Jiao Q. Immunolocalization of native antioxidant scavenger enzymes in early hypertensive and atherosclerotic arteries. Role of oxygen free radicals. *Arterioscler Thromb* 1992; **12**: 403–415.
159. Del Boccio G, Lapenna D, Porreca E, Pennelli A, Savini F, Feliciani P, Ricci G, Cuccurullo F. Aortic antioxidant defence mechanisms: time-related changes in cholesterol-fed rabbits. *Atherosclerosis* 1990; **81**: 127–135.
160. Osborne JA, Lento PH, Siegfried MR, Stahl GL, Fusman B, Lefer AM. Cardiovascular effects of acute hypercholesterolemia in rabbits. Reversal with lovastatin treatment. *J Clin Invest* 1989; **83**: 465-473.
161. Mügge A, Elwell JH, Peterson TE, Hofmeyer TG, Heistad DD, Harrison DG. Chronic treatment with polyethylene-glycolated superoxide dismutase partially restores endothelium-dependent vascular relaxations in cholesterol-fed rabbits. *Circ Res* 1991; **69**: 1293–1300.
162. Keaney JF Jr, Gaziano JM, Xu A, Frei B, Curran-Celentano J, Shwaery GT, Loscalzo J, Vita JA. Low-dose $\alpha$-tocopherol improves and high-dose $\alpha$-tocopherol worsens endothelial vasodilator function in cholesterol-fed rabbits. *J Clin Invest* 1994; **93**: 844–851.
163. Keaney JF Jr, Xu A, Cunningham D, Jackson T, Frei B, Vita JA. Dietary probucol preserves endothelial function in cholesterol-fed rabbits by limiting vascular oxidative stress and superoxide generation. *J Clin Invest* 1995; **95**: 2520–2529.
164. Ohara Y, Peterson TE, Sayegh HS, Subramanian RR, Wilcox JN, Harrison DG. Dietary correction of hypercholesterolemia in the rabbit normalizes endothelial superoxide anion production. *Circulation* 1995; **92**: 898–903.
165. Levine GN, Frei B, Koulouris SN, Gerhard MD, Keaney JF, Vita JA. Ascorbic acid reverses endothelial vasomotor dysfunction in patients with coronary artery disease. *Circulation* 1996; **93**: 1107–1113.
166. McDowell IFW, Brennan GM, McEneny J, Young IS, Nicholls DP, McVeigh GE, Bruce I, Trimble ER, Johnston GD. The effect of probucol and vitamin E treatment on the oxidation of low-density lipoprotein and forearm vascular responses in humans. *Eur J Clin Invest* 1994; **24**: 759–765.
167. Gilligan DM, Sack MN, Guetta V, Casino PR, Quyyumi AA, Rader DJ, Panza JA, Cannon RO. Effect of antioxidant vitamins on low density lipoprotein oxidation and impaired endothelium-dependent vasodilation in patients with hypercholesterolemia. *J Am Coll Cardiol* 1994; **24**: 1611–1617.
168. Marletta MA, Yoon PS, Iyengar R, Leaf CD, Wishnok JS. Macrophage oxidation of L-arginine to nitrite and nitrate: nitric oxide is an intermediate. *Biochemistry* 1988; **27**: 8706–8711.
169. Denis M. Tumor necrosis factor and granulocyte macrophage-colony stimulating factor stimulate human macrophages to restrict growth of virulent *Mycobacterium avium* and to kill avirulent *M. avium*: killing effector mechanism depends on the generation of reactive nitrogen intermediates. *J Leukoc Biol* 1991; **49**: 380–387.
170. Busse R, Mülsch A. Induction of nitric oxide synthase by cytokines in vascular smooth muscle cells. *FEBS Lett* 1990; **275**: 87–90.
171. MacNaul KL, Hutchinson NI. Differential expression of iNOS and cNOS mRNA in human vascular smooth muscle cells and endothelial cells under normal and inflammatory conditions. *Biochem Biophys Res Commun* 1993; **196**: 1330–1334.
172. Berrazueta JR, Salas E, Amado JA, Sanchez de Vega MJ, Poveda JJ. Induction of nitric oxide synthase in human mammary arteries in vitro. *Eur J Pharmacol* 1994; **251**: 303–305.
173. Lamas S, Michel T, Brenner BM, Marsden PA. Nitric oxide synthesis in endothelial cells: evidence for a pathway inducible by TNF-α. *Am J Physiol* 1991; **261**: C634–C641.
174. Radomski MW, Vallance P, Whitley G, Foxwell N, Moncada S. Platelet adhesion to human vascular endothelium is modulated by constitutive and cytokine induced nitric oxide. *Cardiovasc Res* 1993; **27**: 1380–1382.
175. Wood KS, Buga GM, Byrns RE, Ignarro LJ. Vascular smooth muscle-derived relaxing factor (MDRF) and its close similarity to nitric oxide. *Biochem Biophys Res Commun* 1990; **170**: 80–88.
176. Mollace V, Salvemini D, Anggard E, Vane J. Nitric oxide from vascular smooth muscle cells: regulation of platelet reactivity and smooth muscle cell guanylate cyclase. *Br J Pharmacol* 1991; **104**: 633–638.
177. Fleming I, Gray GA, Schott C, Stoclet J-C. Inducible but not constitutive production of nitric oxide by vascular smooth muscle cells. *Eur J Pharmacol* 1991; **200**: 375–376.
178. Schini VB, Busse R, Vanhoutte PM. Inducible nitric oxide synthase in vascular smooth muscle. *Drug Res* 1994; **44**: 432–435.
179. Lang D, Smith JA, Lewis MJ. Induction of a calcium-independent NO synthase by hypercholesterolaemia in the rabbit. *Br J Pharmacol* 1993; **108**: 290–292.
180. Sobey CG, Brooks RM, Heistad DD. Evidence that expression of inducible nitric oxide synthase in response to endotoxin is augmented in atherosclerotic rabbits. *Circ Res* 1995; **77**: 536–543.
181. Russell ME, Wallace AF, Wyner LR, Newell JB, Karnovsky MJ. Upregulation and modulation of inducible nitric oxide synthase in rat cardiac allografts with chronic rejection and transplant arteriosclerosis. *Circulation* 1995; **92**: 457–464.
182. Buttery LDK, Springall DR, Chester AH, Evans TJ, Standfield N, Parums DV, Yacoub MH, Polak JM. Inducible nitric oxide synthase is present within human atherosclerotic lesions and promotes the formation and activity of peroxynitrite. *Lab Invest* 1996; **75**: 77–85.
183. Habib FM, Springall DR, Davies GJ, Oakley CM, Yacoub MH, Polak JM. Tumour necrosis factor and inducible nitric oxide synthase in dilated cardiomyopathy. *Lancet* 1996; **347**: 1151–1155.
184. Wileman SM, Mann GE, Baydoun AR. Induction of L-arginine transport and nitric oxide synthase in vascular smooth muscle cells: synergistic actions of proinflammatory cytokines and bacterial lipopolysaccharide. *Br J Pharmacol* 1995; **116**: 3243–3250.
185. Douglas SA, Vickery-Clark LM, Ohlstein EH. Functional evidence that balloon angioplasty results in transient nitric oxide synthase induction. *Eur J Pharmacol* 1994; **255**: 81–89.
186. Joly GA, Schini VB, Vanhoutte PM. Balloon injury and interleukin-1β induce nitric oxide synthase activity in rat carotid arteries. *Circ Res* 1992; **71**: 331–338.
187. Bosmans JM, Bult H, Vrints CJM, Kockx MM, Claeys M, Snoeck JP, Herman AG. Balloon angioplasty leads to induction of vascular NO-synthase. In: Moncada S, Feelisch M, Busse R, Higgs EA, eds. *The Biology of Nitric Oxide. Volume 3: Physiology and Clinical Aspects*. London: Portland Press, 1994; 34–37.
188. Hansson GK, Geng Y-J, Holm J, Hardhammar P, Wennmalm A, Jennische E. Arterial smooth muscle cells express nitric oxide synthase in response to endothelial injury. *J Exp Med* 1994; **180**: 733–738.
189. Matthys KE, Van Hove CE, Jorens PG, Rosseneu M, Marescau B, Herman AG, Bult H. Dual effects of oxidized low-density lipoprotein on immune-stimulated nitric oxide and prostaglandin release in macrophages. *Eur J Pharmacol* 1996; **298**: 97–103.
190. Pomerantz KB, Hajjar DP, Levi R, Gross SS. Cholesterol enrichment of arterial smooth muscle cells upregulates cytokine-induced nitric oxide synthesis. *Biochem Biophys Res Commun* 1993; **191**: 103–109.
191. Mohan PF, Desaiah D. Very low density and low density lipoproteins induce nitric oxide synthesis in macrophages. *Biochem Biophys Res Commun* 1994; **204**: 1047–1054.
192. Wong HR, Finder JD, Wasserloos K, Pitt BR. Expression of iNOS in cultured rat pulmonary artery smooth muscle cells is inhibited by the heat shock response. *Am J Physiol* 1995; **13**: L843–L848.
193. Colasanti M, Persichini T, Menegazzi M, Mariotto S, Giordano E, Caldarera CM, Sogos V, Lauro GM, Suzuki H. Induction of nitric oxide synthase mRNA expression. Suppression by exogenous nitric oxide. *J Biol Chem* 1995; **270**: 26731–26733.
194. Steinbrecher UP. Role of superoxide in endothelial-cell modification of low-density lipoproteins. *Biochim Biophys Acta* 1988; **959**: 20–30.
195. Rinaldo JE, Clark M, Parinello J, Shepherd VL. Nitric oxide inactivates xanthine dehydrogenase and xanthine oxidase in interferon-gamma-stimulated macrophages. *Am J Respir Cell Mol Biol* 1994; **11**: 625–630.
196. Clancy RM, Leszczynska-Piziak J, Abramson SB. Nitric oxide, an endothelial cell relaxation factor, inhibits neutrophil superoxide anion production via a direct action on the NADPH oxidase. *J Clin Invest* 1992; **90**: 1116–1121.
197. Fukahori M, Ichimori K, Ishida H, Nakagawa H, Okino H. Nitric oxide reversibly suppresses xanthine oxidase activity. *Free Radic Res* 1994; **21**: 203–212.
198. Wink DA, Hanbauer I, Krishna MC, Degraff W, Gamson J, Mitchell JB. Nitric oxide protects against cellular damage and cytotoxicity from reactive oxygen species. *Proc Natl Acad Sci USA* 1993; **21**: 9813–9817.
199. Wink DA, Cook JA, Pacelli R, Liebmann J, Krishna MC, Mitchell JB. Nitric oxide (NO) protects against cellular damage by reactive oxygen species. *Toxicol Lett* 1995; **82**: 221–226.
200. Motterlini R, Foresti R, Intaglietta M, Winslow RM. NO–mediated activation of heme oxygenase: endogenous cytoprotection against oxidative stress to endothelium. *Am J Physiol* 1996; **39**: H107–H114.
201. Jessup W, Mohr D, Gieseg SP, Dean RT, Stocker R. The participation of nitric oxide in cell free- and its restriction of macrophage-mediated oxidation of low-density lipoprotein. *Biochim Biophys Acta* 1992; **1180**: 73–82.
202. Jessup W, Dean RT. Autoinhibition of murine macrophage-mediated oxidation of low-density lipoprotein by nitric oxide synthesis. *Atherosclerosis* 1993; **101**: 145–155.
203. Yates MT, Lambert LE, Whitten JP, McDonald I, Mano M, Ku G, Mao SJT. A protective role for nitric oxide in the oxidative modification of low density lipoproteins by mouse macrophages. *FEBS Lett* 1992; **309**: 135–138.
204. Wang JM, Chow SN, Lin JK. Oxidation of LDL by nitric oxide and its modification by superoxides in macrophage and cell-free systems. *FEBS Lett* 1994; **342**: 171–175.
205. Malo-Ranta U, Ylä-Herttuala S, Metsä-Ketelä T, Jaakkola O, Moilanen E, Vuorinen P, Nikkari T. Nitric oxide donor GEA 3162 inhibits endothelial cell-mediated oxidation of low density lipoprotein. *FEBS Lett* 1994; **337**: 179–183.
206. Rubbo H, Parthasarathy S, Barnes S, Kirk M, Kalyanaraman B, Freeman BA. Nitric oxide inhibition of lipoxygenase-dependent liposome and low-density lipoprotein oxidation: termination of radical chain propagation reactions and formation of nitrogen-containing oxidized lipid derivatives. *Arch Biochem Biophys* 1995; **324**: 15–25.
207. Maccarrone M, Corasaniti MT, Guerrieri P, Nistico G, Agro AF. Nitric oxide-donor compounds inhibit lipoxygenase activity. *Biochem Biophys Res Commun* 1996; **219**: 128–133.
208. Jorens PG, Rosseneu M, Devreese A-M, Bult H, Marescau B, Herman AG. Diminished capacity to release metabolites of nitric oxide synthase in macrophages loaded with oxidized low-density lipoproteins. *Eur J Pharmacol* 1992; **212**: 113–115.
209. Yang X, Cai B, Sciacca RR, Cannon PJ. Inhibition of inducible nitric oxide synthase in macrophages by oxidized low-density lipoproteins. *Circ Res* 1994; **74**: 318–328.
210. Bolton EJ, Jessup W, Stanley KK, Dean RT. Enhanced LDL oxidation by murine macrophage foam cells and their failure to secrete nitric oxide. *Atherosclerosis* 1994; **106**: 213–223.
211. Struck AT, Hogg N, Thomas JP, Kalyanaraman B. Nitric oxide donor compounds inhibit the toxicity of oxidized low-density lipoprotein to endothelial cells. *FEBS Lett* 1995; **361**: 291–294.
212. Suttorp N, Hippenstiel S, Fuhrmann M, Krull M, Podzuweit T. Role of nitric oxide and phosphodiesterase isoenzyme II for reduction of endothelial hyperpermeability. *Am J Physiol* 1996; **39**: C778–C785.
213. Niu X-F, Smith CW, Kubes P. Intracellular oxidative stress induced by nitric oxide synthase inhibition increases endothelial cell adhesion to neutrophils. *Circ Res* 1994; **74**: 1133–1140.
214. Mehta A, Yang B, Khan S, Hendricks JB, Stephen C, Mehta JL. Oxidized low-density lipoproteins facilitate leukocyte adhesion to aortic intima without affecting endothelium-dependent relaxation. Role of P-selectin. *Arterioscler Thromb Vasc Biol* 1995; **15**: 2076–2083.
215. Ma X-L, Weyrich AS, Lefer DJ, Lefer AM. Diminished basal nitric oxide release after myocardial ischemia and reperfusion promotes neutrophil adherence to coronary endothelium. *Circ Res* 1993; **72**: 403–412.
216. Siegfried MR, Carey C, Ma X-L, Lefer AM. Beneficial effects of SPM-5185, a cysteine-containing NO donor in myocardial ischemia-reperfusion. *Am J Physiol* 1992; **263**: H771–H777.
217. De Caterina R, Libby P, Peng HB, Thannickal VJ, Rajavashisth TB, Gimbrone MA Jr, Shin WS, Liao JK. Nitric oxide decreases cytokine-induced endothelial activation. Nitric oxide selectively reduces endothelial expression of adhesion molecules and proinflammatory cytokines. *J Clin Invest* 1995; **96**: 60–68.

218. Zeiher AM, Fisslthaler B, Schray-Utz B, Busse R. Nitric oxide modulates the expression of monocyte chemoattractant protein 1 in cultured human endothelial cells. *Circ Res* 1995; **76**: 980–986.
219. Bath PMW, Hassall DG, Gladwin A-M, Palmer RMJ, Martin JF. Nitric oxide and prostacyclin: divergence of inhibitory effects on monocyte chemotaxis and adhesion to endothelium in vitro. *Arterioscler Thromb* 1991; **11**: 254–260.
220. Tsao PS, Lewis NP, Alpert S, Cooke JP. Exposure to shear stress alters endothelial adhesiveness. Role of nitric oxide. *Circulation* 1995; **92**: 3513–3519.
221. Scott-Burden T, Vanhoutte PM. The endothelium as a regulator of vascular smooth muscle proliferation. *Circulation* 1993; **87 (suppl V)**: V-51–V-55.
222. Mooradian DL, Hutsell TC, Keefer LK. Nitric oxide (NO) donor molecules: effect of NO release rate on vascular smooth muscle cell proliferation in vitro. *J Cardiovasc Pharmacol* 1995; **25**: 674–678.
223. Assender JW, Southgate KM, Newby AC. Does nitric oxide inhibit smooth muscle proliferation? *J Cardiovasc Pharmacol* 1991; **17 (suppl 3)**: S104–S107.
224. Garg UC, Hassid A. Nitric oxide-generating vasodilators and 8-bromo-cyclic guanosine monophosphate inhibit mitogenesis and proliferation of cultured rat vascular smooth muscle cells. *J Clin Invest* 1989; **83**: 1774–1777.
225. Cornwell TL, Arnold E, Boerth NJ, Lincoln TM. Inhibition of smooth muscle cell growth by nitric oxide and activation of cAMP-dependent protein kinase by cGMP. *Am J Physiol* 1994; **36**: C1405–C1413.
226. Nakaki T, Nakayama M, Kato R. Inhibition by nitric oxide and nitric oxide-producing vasodilators of DNA synthesis in vascular smooth muscle cells. *Eur J Pharmacol* 1990; **189**: 347–353.
227. Kariya K, Kawahara Y, Araki S, Fukuzaki H, Takai Y. Antiproliferative action of cyclic GMP-elevating vasodilators in cultured rabbit aortic smooth muscle cells. *Atherosclerosis* 1989; **80**: 143–147.
228. Sarkar R, Meinberg EG, Stanley JC, Gordon D, Webb RC. Nitric oxide reversibly inhibits the migration of cultured vascular smooth muscle cells. *Circ Res* 1996; **78**: 225–230.
229. Dubey RK, Jackson EK, Lüscher TF. Nitric oxide inhibits angiotensin II-induced migration of rat aortic smooth muscle cell. *J Clin Invest* 1995; **96**: 141–149.
230. Fu Y, Blankenhorn EP. Nitric oxide-induced anti-mitogenic effects in high and low responder rat strains. *J Immunol* 1992; **148**: 2217–2222.
231. Kawabe T, Isobe KI, Hasegawa Y, Nakashima I, Shimokata K. Immunosuppressive activity induced by nitric oxide in culture supernatant of activated rat alveolar macrophages. *Immunology* 1992; **76**: 72–78.
232. Merryman PF, Clancy RM, He XY, Abramson SB. Modulation of human T-cell responses by nitric oxide and its derivative, S-nitrosoglutathione. *Arthritis and Rheumatism* 1993; **36**: 1414–1422.
233. Morbidelli L, Chang CH, Douglas JG, Granger HJ, Ledda F, Ziche M. Nitric oxide mediates mitogenic effect of VEGF on coronary venular endothelium. *Am J Physiol* 1996; **39**: H411–H415.
234. Schafer AI, Alexander RW, Handin RI. Inhibition of platelet function by organic nitrate vasodilators. *Blood* 1980; **55**: 649–654.
235. Radomski MW, Palmer RMJ, Moncada S. Comparative pharmacology of endothelium-derived relaxing factor, nitric oxide and prostacyclin in platelets. *Br J Pharmacol* 1987; **92**: 181–187.
236. Bassenge E. Antiplatelet effects of endothelium-derived relaxing factor and nitric oxide donors. *Eur Heart J* 1991; **12 (suppl E)**: 12–15.
237. Cooke JP, Singer AH, Tsao P, Zera P, Rowan RA, Billingham ME. Antiatherogenic effects of L-arginine in the hypercholesterolemic rabbit. *J Clin Invest* 1992; **90**: 1168–1172.
238. Tsao PS, McEvoy LM, Drexler H, Butcher EC, Cooke JP. Enhanced endothelial adhesiveness in hypercholesterolemia is attenuated by L-arginine. *Circulation* 1994; **89**: 2176–2182.
239. Kubes P, Suzuki M, Granger DN. Nitric oxide: an endogenous modulator of leukocyte adhesion. *Proc Natl Acad Sci USA* 1991; **88**: 4651–4655.
240. Gaboury J, Woodman RC, Granger DN, Reinhardt P, Kubes P. Nitric oxide prevents leukocyte adherence: role of superoxide. *Am J Physiol* 1993; **265**: H862–H867.
241. Gauthier TW, Davenpeck KL, Lefer AM. Nitric oxide attenuates leukocyte endothelial interaction via P-selectin in splanchnic ischemia-reperfusion. *Am J Physiol* 1994; **30**: G562–G568.
242. Kojda G, Noack E. Effects of pentaerythrityl-tetranitrate and isosorbide-5-mononitrate in experimental atherosclerosis. *Agents Actions Suppl* 1995; **45**: 201–206.
243. Naruse K, Shimizu K, Muramatsu M, Toki Y, Miyazaki Y, Okumura K, Hashimoto H, Ito T. Long-term inhibition of NO synthesis promotes atherosclerosis in the hypercholesterolemic rabbit thoracic aorta. *Arterioscler Thromb* 1994; **14**: 746–752.
244. Cayatte AJ, Palacino JJ, Horten K, Cohen RA. Chronic inhibition of nitric oxide production accelerates neointima formation and impairs endothelial function in hypercholesterolemic rabbits. *Arterioscler Thromb* 1994; **14**: 753–759.
245. Lüscher TF, Boulanger CM, Yang Z, Noll G, Dohi Y. Interactions between endothelium-derived relaxing and contracting factors in health and cardiovascular disease. *Circulation* 1993; **87 (suppl V)**: V-36–V-44.
246. Lüscher TF. Endothelium in the control of vascular tone and growth: role of local mediators and mechanical forces. *Blood Press* 1994; **(suppl 1)**: p18–p22.
247. Rizvi MAD, Katwa L, Spadone DP, Myers PR. The effects of endothelin-1 on collagen type I and type III synthesis in cultured porcine coronary artery vascular smooth muscle cells. *J Mol Cellul Cardiol* 1996; **28**: 243–252.
248. Lerman A, Edwards BS, Hallett JW, Heublein DM, Sandberg SM, Burnett JC. Circulating and tissue endothelin immunoreactivity in advanced atherosclerosis. *New Engl J Med* 1991; **325**: 997–1001.
249. Davies MG, Dalen H, Austarheim AMS, Gulbrandsen TF, Svendsen E, Hagen PO. Suppression of intimal hyperplasia in experimental vein grafts by oral L-arginine supplementation and single ex vivo immersion in deferoxamine manganese. *J Vasc Surg* 1996; **23**: 410–420.
250. McNamara DB, Bedi B, Aurora H, Ignarro LJ, Kadowitz PJ, Akers DL. L-arginine inhibits balloon cathether-induced intimal hyperplasia. *Biochem Biophys Res Commun* 1993; **193**: 291–296.
251. Taguchi J, Abe J, Okazaki H, Takuwa Y, Kurokawa K. L-arginine inhibits neointimal formation following balloon injury. *Life Sci* 1993; **53**: 387–392.
252. Ellenby MI, Ernst CB, Carretero OA, Scicli AG. Role of nitric oxide in the effect of blood flow on neointima formation. *J Vasc Surg* 1996; **23**: 314–422.
253. Fahry RD, Carretero OA, Ho KL, Scicli AG. Role of kinins and nitric oxide in the effects of angiotensin converting enzyme inhibitors on neointima formation. *Circ Res* 1993; **72**: 1202–1210.
254. Van Belle E, Vallet B, Auffray JL, Bauters C, Hamon M, McFadden EI, Lablanche JM, Dupuis E, Bertrand ME. NO synthesis is involved in structural and functional effects of ACE inhibitors in injured arteries. *Am J Physiol* 1996; **39**: H298–H305.
255. Guo J-P, Milhoan KA, Tuan RS, Lefer AM. Beneficial effect of SPM-5185, a cysteine-containing nitric oxide donor, in rat carotid artery intimal injury. *Circ Res* 1994; **75**: 77–84.
256. Lee JS, Adrie C, Jacob HJ, Roberts JD Jr, Zapol WM, Bloch KD. Chronic inhalation of nitric oxide inhibits neointimal formation after balloon-induced arterial injury. *Circ Res* 1996; **78**: 337–342.
257. Wolf YG, Rasmussen LM, Sherman Y, Bundens WP, Hye RJ. Nitroglycerin decreases medial smooth muscle cell proliferation after arterial balloon injury. *J Vasc Surg* 1995; **21**: 499–504.
258. Marks DS, Vita JA, Folts JD, Keaney JF, Welch GN, Loscalzo J. Inhibition of neointimal proliferation in rabbits after vascular injury by a single treatment with a protein adduct of nitric oxide. *J Clin Invest* 1995; **96**: 2630–2638.
259. von der Leyen HE, Gibbons GH, Morishita R, Lewis NP, Zhang L, Nakajima M, Kaneda Y, Cooke JP, Dzau VJ. Gene therapy inhibiting neointimal vascular lesion: in vivo transfer of endothelial cell nitric oxide synthase gene. *Proc Natl Acad Sci USA* 1995; **92**: 1137–1141.
260. De Meyer GRY, Bult H, Üstünes L, Kockx MM, Feelisch M, Herman AG. Effect of nitric oxide donors on neointima formation and vascular reactivity in the collared carotid artery of rabbits. *J Cardiovasc Pharmacol* 1995; **26**: 272–279.
261. FitzGerald GA. Dipyridamole. *N Engl J Med* 1987; **316**: 1247–1257.
262. Groves PH, Banning AP, Penny WJ, Newby AC, Cheadle HA, Lewis MJ. The effects of exogenous nitric oxide on smooth muscle cell proliferation following porcine carotid angioplasty. *Cardiovasc Res* 1995; **30**: 87–96.
263. Chang GJ, Woo P, Honda HM, Ignarro LJ, Young L, Berliner JA, Demer LL. Oxidation of LDL to a biologically active form by derivatives of nitric oxide and nitrite in the absence of superoxide. Dependence on pH and oxygen. *Arterioscler Thromb* 1994; **14**: 1808–1814.
264. Darley-Usmar VM, Hogg N, O'Leary VJ, Wilson MT, Moncada S. The simultaneous generation of superoxide and nitric oxide can initiate lipid peroxidation in human low density lipoprotein. *Free Rad Res Comms* 1992; **17**: 9–20.
265. Hogg N, Darley-Usmar VM, Wilson MT, Moncada S. The oxidation of alpha-tocopherol in human low-density lipoprotein by the simultaneous generation of superoxide and nitric oxide. *FEBS Lett* 1993; **326**: 199–203.
266. de Groot H, Hegi U, Sies H. Loss of alpha-tocopherol upon exposure to nitric oxide or the sydnonimine SIN-1. *FEBS Lett* 1993; **315**: 139–142.
267. Graham A, Hogg N, Kalyanaraman B, O'Leary V, Darley-Usmar VM, Moncada S. Peroxynitrite modification of low-density lipoprotein leads to recognition by the macrophage scavenger receptor. *FEBS Lett* 1993; **330**: 181–185.
268. Beckman JS, Beckman TW, Chen J, Marshall PA, Freeman BA. Apparent hydroxyl radical production by peroxynitrite: implications for endothelial injury from nitric oxide and superoxide. *Proc Natl Acad Sci USA* 1990; **87**: 1620–1624.
269. Radi R, Beckman JS, Bush KM, Freeman BA. Peroxynitrite oxidation of sulfhydryls. The cytotoxic potential of superoxide and nitric oxide. *J Biol Chem* 1991; **266**: 4244–4250.
270. Beckmann JS, Ye YZ, Anderson PG, Chen J, Accavitti MA, Tarpey MM, White CR. Extensive nitration of protein tyrosines in human atherosclerosis detected by immunohistochemistry. *Biol Chem Hoppe-Seyler* 1994; **375**: 81–88.

271. Maruyama W, Hashizume Y, Matsubara K, Naoi M. Identification of 3-nitro-L-tyrosine, a product of nitric oxide and superoxide, as an indicator of oxidative stress in the human brain. *J Chromatography* 1996; **676**: 153-158.
272. Palmer RM, Bridge L, Foxwell NA, Moncada S. The role of nitric oxide in endothelial cell damage and its inhibition by glucocorticoids. *Br J Pharmacol* 1992; **105**: 11–12.
273. Fukuo K, Inoue T, Morimoto S, Nakahashi T, Yasuda O, Kitano S, Sasada R, Ogihara T. Nitric oxide mediates cytotoxicity and basic fibroblast growth factor release in cultured vascular smooth muscle cells. *J Clin Invest* 1995; **95**: 669–676.
274. Szabo C, Zingarelli B, O'Connor M, Salzman AL. DNA strand breakage, activation of poly(ADP-ribose) synthetase, and cellular energy depletion are involved in the cytotoxicity in macrophages and smooth muscle cells exposed to peroxynitrite. *Proc Natl Acad Sci USA* 1996; **93**: 1753–1758.
275. Forrester K, Ambs S, Lupold SE, Kapust RB, Spillare EA, Weinberg WC, Felley Bosco E, Wang XW, Geller DA, Tzeng E, Billiar TR, Harris CC. Nitric oxide-induced p53 accumulation and regulation of inducible nitric oxide synthase expression by wild-type p53. *Proc Natl Acad Sci USA* 1996; **93**: 2442–2447.
276. Albina JE, Cui S, Mateo RB, Reichner JS. Nitric oxide-mediated apoptosis in murine peritoneal macrophages. *J Immunol* 1993; **150**: 5080–5085.
277. Sarih M, Souvannavong V, Adam A. Nitric oxide synthase induces macrophage death by apoptosis. *Biochem Biophys Res Commun* 1993; **191**: 503–508.
278. Fukuo K, Hata S, Suhara T, Nakahashi T, Shinto Y, Tsujimoto Y, Morimoto S, Ogihara T. Nitric oxide induces upregulation of Fas and apoptosis in vascular smooth muscle. *Hypertension* 1996; **27**: 823–826.
279. Bochaton-Piallat M-L, Gabbiani F, Redard M, Desmoulière A, Gabbiani G. Apoptosis participates in cellularity regulation during rat aortic intimal thickening. *Am J Pathol* 1995; **146**: 1059–1064.
280. Isner JF, Kearney M, Bortman S, Passeri J. Apoptosis in human atherosclerosis and restenosis. *Circulation* 1995; **91**: 2703–2711.
281. Kockx MM, De Meyer GRY, Muhring J, Bult H, Bultinck J, Herman AG. Distribution of cell replication and apoptosis in atherosclerotic plaques of cholesterol-fed rabbits. *Atherosclerosis* 1996; **120**: 115–124.
282. Trachtman H, Futterweit S, Garg P, Reddy K, Singhai PC. Nitric oxide stimulates the activity of a 72-kDa neutral matrix metalloproteinase in cultured rat mesangial cells. *Biochem Biophys Res Commun* 1996; **218**: 704–708.
283. Murrell GAC, Jang D, Williams RJ. Nitric oxide activates metalloprotease enzymes in articular cartilage. *Biochem Biophys Res Commun* 1995; **206**: 15–21.
284. Frears ER, Zhang Z, Blake DR, O'Connell JP, Winyard PG. Inactivation of tissue inhibitor of metalloproteinase-1 by peroxynitrite. *FEBS Lett* 1996; **381**: 21–24.
285. Miller MJS, Grisham MB. Nitric oxide as a mediator of inflammation? You had better believe it. *Med Inflammation* 1995; **4**: 387–396.
286. Messmer UK, Brune B. Nitrix oxide (NO) in apoptotic versus necrotic RAW 264.7 macrophage cell death: the role of NO-donor exposure, NAD(+) content, and p53 accumulation. *Arch Biochem Biophys* 1996; **327**: 1–10.

ACKNOWLEDGEMENTS. The authors wish to thank Ms L. Van Den Eynde for secretarial assistance. The work was supported by the Belgian Programme on Interuniversity Poles of Attraction, Prime Minister's Office, Science Policy Planning.

**Received 10 July 1996**
**accepted in revised form 29 August 1996**
